# Dissecting Bottromycin Biosynthesis Using Comparative Untargeted Metabolomics

**DOI:** 10.1002/anie.201604304

**Published:** 2016-07-04

**Authors:** William J. K. Crone, Natalia M. Vior, Javier Santos‐Aberturas, Lukas G. Schmitz, Finian J. Leeper, Andrew W. Truman

**Affiliations:** ^1^ Department of Molecular MicrobiologyJohn Innes CentreColney LaneNorwichNR4 7UHUK; ^2^ Department of ChemistryUniversity of CambridgeLensfield RoadCambridgeCB2 1EWUK

**Keywords:** biosynthesis, bottromycin, mass spectrometry, natural products, peptides

## Abstract

Bottromycin A_2_ is a structurally unique ribosomally synthesized and post‐translationally modified peptide (RiPP) that possesses potent antibacterial activity towards multidrug‐resistant bacteria. The structural novelty of bottromycin stems from its unprecedented macrocyclic amidine and rare β‐methylated amino acid residues. The *N*‐terminus of a precursor peptide (BtmD) is converted into bottromycin A_2_ by tailoring enzymes encoded in the *btm* gene cluster. However, little was known about key transformations in this pathway, including the unprecedented macrocyclization. To understand the pathway in detail, an untargeted metabolomic approach that harnesses mass spectral networking was used to assess the metabolomes of a series of pathway mutants. This analysis has yielded key information on the function of a variety of previously uncharacterized biosynthetic enzymes, including a YcaO domain protein and a partner protein that together catalyze the macrocyclization.

Ribosomally synthesized and post‐translationally modified peptides (RiPPs) are natural products that are prevalent throughout nature,^1^ and their biosynthetic pathways are capable of transforming simple proteinogenic amino acids into structurally complex compounds that have potent bioactivities.^2^, ^3^, ^4^ However, elucidating the biosynthesis of RiPPs can be hindered by the difficulty of isolating intermediates, as the biosynthesis takes place on a larger precursor peptide, and intermediates may be rapidly proteolyzed. Therefore, improved methods for the identification of RiPP intermediates are desirable. Bottromycin A_2_ (**1**, Scheme 1)^5^, ^6^, ^7^, ^8^ possesses potent antibacterial activity towards multidrug‐resistant bacteria,^9^ and is structurally unique owing its unprecedented macrocyclic amidine, rare β‐methylated amino acids residues, and a terminal thiazole. Nature employs a variety of strategies for peptide macrocyclization,^10^, ^11^, ^12^ but amidine formation has only been observed for bottromycin. Initial studies on bottromycin biosynthesis showed that its amino acids were β‐methylated by radical SAM methyltransferases^5^, ^7^ (RSMTs), but the rest of the bottromycin pathway represented a biosynthetic black box, where little was known about key steps in the pathway, including the unprecedented macrocyclization. In this study, we employ untargeted metabolomics and mass spectral networking to deduce the biosynthetic route to bottromycins in *Streptomyces scabies*. This analysis identifies the enzymes responsible for macrocyclization, thiazole formation, and aspartate epimerization, thereby demonstrating the utility of an untargeted metabolomic approach for elucidating a targeted biosynthetic pathway.

**Scheme 1:**
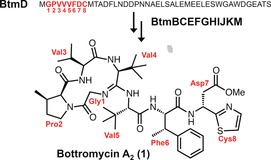
Conversion of BtmD into bottromycin A_2_ (**1**).

To assess the role of the putative tailoring genes in the bottromycin pathway (Supporting Information, Figure S1), we had previously generated *S. scabies ΔbtmC*, *ΔbtmE*, *ΔbtmF*, *ΔbtmI*, and *ΔbtmJ*, but were unable to identify bottromycin‐like compounds in these mutants.^5^ We therefore established that these deletions did not lead to deleterious polar effects on the pathway by successfully complementing each mutant strain with a copy of the deleted gene (Supporting Information, Table S2 and Figure S2). Furthermore, an RT‐PCR analysis of wild‐type and *ΔbtmD* strains showed that transcription still occurs in the absence of the precursor peptide at a comparable level to the wild‐type (WT) strain (Supporting Information, Figure S3), indicating that there is no essential regulatory feedback mechanism associated with the production of a pathway intermediate.^13^

Therefore, a comparative untargeted metabolomic analysis was carried out using WT *S. scabies* alongside the *ΔbtmC*, *ΔbtmD*, *ΔbtmE*, *ΔbtmF*, *ΔbtmG*, *ΔbtmI*, and *ΔbtmJ* deletion strains. Untargeted metabolomics is frequently used to assess the total metabolome of an organism,^14^ for example to prioritize strains and compounds for drug discovery,^15^, ^16^ or to identify novel natural products,^17^ but has rarely been used to assess a single pathway. High‐resolution liquid chromatography–mass spectrometry (LC‐MS) data for triplicate three‐day production cultures of each strain (Supporting Information, Figure S4) were analyzed using two untargeted comparative metabolic methods. First, global LC‐MS metabolomic profiles for each strain were used to generate an aligned data matrix that indicated significant differences between each mutant (Supporting Information, Table S5). This dataset was filtered to remove any metabolites that appeared in either *ΔbtmD* or the production medium. Mature bottromycins (**1**–**5**, Figure 2) were clearly absent in every mutant, but the complexity of the data hampered the detailed characterization of metabolites. Therefore, this was followed by mass spectral network analysis,^18^ which is a powerful tool that identifies similarities in MS^2^ fragmentation data and builds a network of species with related MS^2^ spectra, thus identifying structurally‐related molecules within a complex mixture.^18^, ^19^, ^20^, ^21^, ^22^, ^23^, ^24^ This has been used to assess the global metabolic profiles of a single organism, either in isolation^19^ or when interacting with neighboring species,^18^ to compare the metabolomes of related organisms,^20^, ^21^, ^22^ to assess the metabolic potential of a new bacterial taxon,^23^ and to identify metabolites related to the colibactin pathway.^24^

Mass spectral network analysis of WT, *ΔbtmC*, *ΔbtmD*, *ΔbtmF*, *ΔbtmI*, and *ΔbtmJ* strains revealed an extensive metabolic network (Supporting Information, Figure S5). An analysis of the metabolomes of *ΔbtmE* and *ΔbtmG* was used to map molecules produced by these mutants onto this network. Nodes representing species that were not present in *ΔbtmD* were manually assessed using MS^2^ to identify molecules related to the *btm* pathway. This global metabolomic analysis showed that the bottromycin pathway contributes much more to the total metabolite profile of *S. scabies* than was previously understood,^25^ and identified 14 distinct molecules in the wild‐type strain, and at least 6 additional molecules across the mutant strains, with masses and fragmentation patterns that are entirely consistent with bottromycin‐like molecules (**1**–**20**; Figure 1, Figure 2; Supporting Information, Figures S6–S23, Table S3). The only significant molecule that was not revealed by network analysis, owing to a lack of MS^2^ fragmentation homology, was an abundant species with *m*/*z* 406.27 (**17**; Figure 2; Supporting Information, Figure S17), which was identified by the initial comparative analysis of LC‐MS data. The abundance of various bottromycin‐like metabolites in WT *S. scabies* (Figures 1; Supporting Information, Figure S23) indicates that there are significant bottlenecks in the biosynthetic pathway that preclude the efficient processing of BtmD into bottromycin. Instead, partially processed BtmD can be proteolyzed, and the data show that there are multiple points at which the pathway stalls. The diversity of bottromycin‐like molecules produced by the WT could explain why it was difficult in prior studies to identify novel metabolites from mutants.

**Figure 1:**
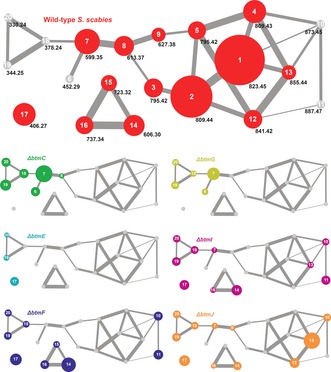
Bottromycin mass spectral network from WT *S. scabies* and a series of pathway mutants. Each node represents one metabolite and edge thickness between nodes reflects the relative similarity of MS^2^ data. The WT network is annotated with all observed *m*/*z* data and is enlarged ×2 for clarity. Gray nodes indicate an absence of a compound and the area of the node is partially proportional to the abundance of the metabolite.

**Figure 2:**
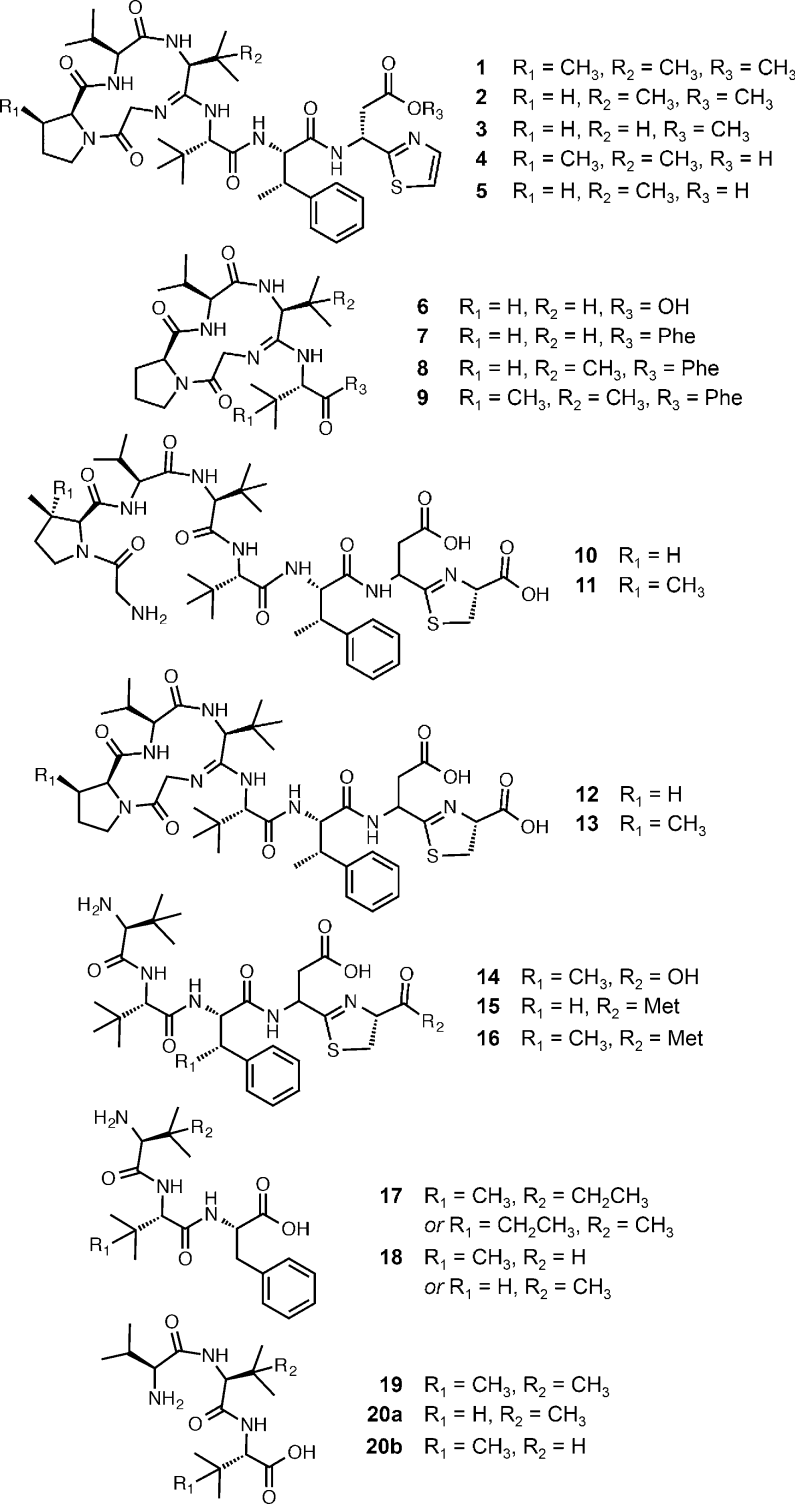
Bottromycin pathway metabolites identified in this study. Predicted stereochemistry is based on the ribosomal origin of each amino acid, although Asp 7 stereochemistry is not provided for some compounds owing to the potential for epimerization and corresponding double peaks in their LC traces.

The macrocyclic amidine of bottromycin is unique in nature, and a plausible biosynthetic route involves the nucleophilic attack of Gly 1 onto the amide bond between Val 4 and Val 5, which could require the activation of the amide carbonyl. YcaO domain proteins activate backbone amide bonds by phosphorylation^26^, ^27^ or adenylation^28^ of the carbonyl oxygen, and all YcaO domain proteins with a characterized activity have a partner cyclodehydratase that aids catalysis of cyclization to oxazolines or thiazolines.^29^ The bottromycin gene cluster encodes two YcaO domain proteins, BtmE and BtmF, but no cyclodehydratases. Therefore, we hypothesized that one participates in macrocyclization and the other is involved in the formation of the terminal thiazole.

Analysis of the comparative metabolomic and MS^2^ network datasets revealed two new molecules (*m*/*z* 873.45 and *m*/*z* 887.47) produced by both *ΔbtmF* and the amidohydrolase mutant *ΔbtmI*, but not by WT *S. scabies*. Masses of 873.45 Da and 887.47 Da correspond to the addition of H_2_O to carboxylated *O*‐desmethylated bottromycins A_2_ (**1**) and C_2_,^25^ respectively, which indicated that one of the cyclodehydrations does not occur in *ΔbtmF* and *ΔbtmI*. MS^n^ revealed that these molecules are not macrocyclized but do feature the thiazoline ring (**10** and **11**; Figure 3 a; Supporting Information, Figure S10), thus indicating that BtmF and BtmI cooperate to catalyze amidine ring formation, but are not required for thiazoline formation. Both mutant strains also produced a range of other bottromycin derivatives that contain a thiazoline ring but no macrocycle (Figure 1, Figure 2; Supporting Information, Figure S23). *ΔbtmI* did produce trace amounts of macrocyclized **7** and **13**, which could reflect inefficient spontaneous cyclization following BtmF‐catalyzed amide activation. A cyclization mechanism is proposed (Figure 3 b) where BtmF activates the amide bond using ATP and BtmI catalyzes cyclization. Further experiments with purified proteins will be needed to verify this, especially in relation to timing of ATP activation. Cyclization is contingent on the removal of the *N*‐terminal methionine, which is usually catalyzed by endogenous aminopeptidases, but these do not function efficiently with an MGP sequence.^30^ In vitro analysis of the M17 peptidase^31^ BtmM with BtmD demonstrated that BtmM catalyzes this reaction when either Zn^2+^ or Co^2+^ are used as co‐factors (Supporting Information, Figures S25 and S26).

**Figure 3:**
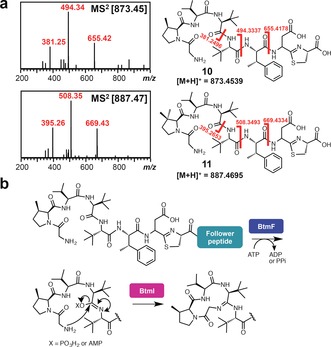
Macrocyclization catalyzed by BtmF and BtmI. a) Characterization of **10** and **11** produced by *S. scabies ΔbtmF* and *ΔbtmI*. Further MS^2^ and MS^3^ characterization is shown in the Supporting Information, Figure S10. b) Proposed macrocyclization mechanism.

In contrast to *ΔbtmF*, the only abundant species that could be confidently assigned as a BtmD‐derived metabolite in *ΔbtmE* was **17** (Supporting Information, Figure S16), which is a trimethylated tripeptide that is also found in the WT, *ΔbtmF*, *ΔbtmI*, and *ΔbtmJ* strains (Supporting Information, Figure S23). This assignment is consistent with the absence of **17** in *ΔbtmD* and in the RSMT mutants *ΔbtmC* and *ΔbtmG*. Unfortunately, this provided no evidence on Cys8 cyclization; the absence of cysteine‐containing peptides could reflect rapid peptide degradation when cyclization does not occur. The lack of any thiazole or thiazoline‐containing metabolites does imply that BtmE catalyzes thiazoline formation, although further in vitro characterization is required to confirm this. The absence of macrocyclized metabolites suggests that thiazoline formation is an early step in the pathway. BtmH, the only uncharacterized hydrolytic enzyme in the pathway, is proposed to remove the follower peptide, although it is possible that it could also participate in heterocyclization.

The *btm* cluster lacks a flavin‐dependent dehydrogenase that is required for the biosynthesis of all other thiazole/oxazole‐containing RiPPs.^29^ Instead, a P450 enzyme, BtmJ, was predicted to catalyze the oxidative decarboxylation of the thiazoline into a thiazole.^5^, ^6^, ^7^ This is an uncommon role for a P450, although it has been reported for thiazole formation in the biosynthesis of the plant alkaloid camalexin^32^ and could be mechanistically similar to the fatty acid P450 decarboxylase OleT.^33^ Analysis of *ΔbtmJ* revealed two abundant compounds with *m*/*z* 841.43 and 855.44 (Figure 4), which were confirmed to be carboxylated *O*‐desmethyl bottromycins B_2_ and A_2_, respectively (**12** and **13**) using MS^2^ (Supporting Information, Figure S11).

**Figure 4:**
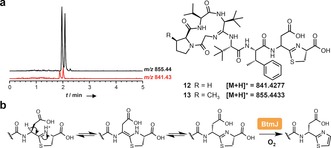
BtmJ‐catalyzed oxidative decarboxylation. a) LC‐MS spectra showing double peaks for **12** and **13** identified in *S. scabies ΔbtmJ*. b) Mechanistic proposal for epimerization followed by oxidation.

Interestingly, two distinct peaks are observed by LC‐MS for both *m*/*z* 855.44 and 841.43 (Figure 4 a), and each pair of peaks with the same mass have identical MS^2^ fragmentation patterns (Supporting Information, Figure S11). This could reflect a mixture of epimers at the aspartate residue, which has a non‐proteinogenic d‐stereocenter in bottromycin A_2_. Therefore, we hypothesized that aspartate epimerization occurs after thiazoline formation, when the p*K*_a_ of the aspartate α‐proton is lowest owing to imine–enamine tautomerization that is disfavored once the aromatic thiazole is formed (Figure 4 b). This is consistent with previous reports of epimerization of amino acids adjacent to carboxylated thiazolines,^34^ and we could observe spontaneous interconversion of these peaks at pH 7.5 (Supporting Information, Figure S12).

To further assess whether this proton is exchangeable, we carried out a deuterium labeling experiment. Here, all exchangeable protons were replaced with deuterium in D_2_O, the thiazoline was then hydrolyzed back to Cys in dilute aq. DCl, and the sample was finally treated with H_2_O. Theoretically, this would trap a deuterium in the Asp α‐position as back exchange would be prevented following loss of the thiazoline. This indeed showed specific incorporation of one deuterium into **13** at Asp 7 (Supporting Information, Figures S13 and S14), indicating that this position can readily undergo non‐enzymic epimerization. The drop in abundance of both forms of *m*/*z* 855.44 in the WT compared to *ΔbtmJ* (Supporting Information, Figure S23) implies that a dynamic kinetic resolution converts this mixture of epimers into stereochemically pure mature bottromycins.

To investigate whether any of the metabolites reported are authentic pathway intermediates, each mutant strain was co‐cultivated with *ΔbtmD*, which is unable to produce the precursor peptide. Any diffusible molecules produced by mutants that are genuine intermediates should be converted into **1** by the functional enzymes in *ΔbtmD*. Only the *ΔbtmJ*+*ΔbtmD* co‐cultivation resulted in the production of **1** (Supporting Information, Figure S27), which implies that **12** and **13** are true intermediates and supports the proposed roles and substrate specificities of BtmJ and BtmB. In contrast, the failure of the *ΔbtmF* and *ΔbtmI* co‐cultivation experiments suggests that the linear compounds **10** and **11** are shunt metabolites rather than authentic intermediates, and that BtmF and BtmI require a substrate that contains a follower peptide. However, we cannot rule out the possibility that **10**/**11** are not exported/imported as effectively as **12**/**13**. The lack of an O‐methyl group on the d‐aspartyl residue in any of the metabolites identified from mutant strains indicates that O‐methylation is the last step in the pathway, thereby generating an active antibiotic.^35^ This was supported by the in vitro O‐methylation of **4** using recombinant BtmB (Supporting Information, Figure S28).

Three RSMTs catalyze four C‐methylations in the *btm* pathway.^5^, ^7^ In *S. scabies*, bottromycin production is either severely reduced or entirely abolished when either RSMT gene, *btmC* or *btmG*, is deleted. BtmG methylates Val 4 and Val 5, and BtmC methylates Phe 6,^5^, ^7^ but it is unclear why the pathway stalls when either step is missed. The metabolic datasets showed that both *ΔbtmC* and *ΔbtmG* have highly similar metabolite profiles, and the production of macrocyclized shunt metabolites **6** and **7** indicates that C‐methylation is not a prerequisite for cyclization. However, the fully C‐methylated metabolites produced by *ΔbtmF* and *ΔbtmI* demonstrate that macrocyclization is not a prerequisite for C‐methylation either. Also, the production of methylated tripeptides by *ΔbtmC* and *ΔbtmG* indicates that the pathway can stall before cyclization when *C*‐methylation is disrupted. The data are consistent with incomplete C‐methylation reducing the efficiency of various downstream modification steps.

There has been widespread recent interest in both the biosynthesis^5^, ^6^, ^7^, ^8^ and biological activity^9^, ^35^ of bottromycin owing to its unusual structure and potent antimicrobial activity. In this study, we have harnessed untargeted metabolomics to elucidate the biosynthetic pathway to bottromycin A_2_ (Scheme 2; Supporting Information, Table S4). Our analysis identified a wide array of metabolites related to bottromycin, and the untargeted metabolomic data matrix (Supporting Information, Table S5) indicates that there may be further, currently uncharacterized, metabolites produced by this pathway. This study also reveals the first example of YcaO domain‐catalyzed macrocyclization, which provides the foundation for detailed mechanistic investigations into this step.

**Scheme 2:**
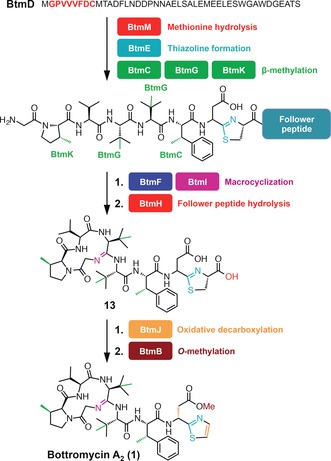
Revised bottromycin pathway.

## Supporting information

As a service to our authors and readers, this journal provides supporting information supplied by the authors. Such materials are peer reviewed and may be re‐organized for online delivery, but are not copy‐edited or typeset. Technical support issues arising from supporting information (other than missing files) should be addressed to the authors.

Supplementary
