# Supplementary material for: Dissecting Bottromycin Biosynthesis Using Comparative Untargeted Metabolomics
Source: Angew Chem Int Ed Engl. 2016 Jul 4;55(33):9639–43. doi: 10.1002/anie.201604304 (PMC5103208; doi:10.1002/anie.201604304)
Supplement: Supplementary file 1 — Supplementary [file ANIE-55-9639-s001.pdf]

Angewandte Chemie
Eine Zeitschrift der Gesellschaft Deutscher Chemiker

Supporting Information

Dissecting Bottromycin Biosynthesis Using Comparative Untargeted Metabolomics

*William J. K. Crone, Natalia M. Vior, Javier Santos-Aberturas, Lukas G. Schmitz, Finian J. Leeper, and Andrew W. Truman**

anie_201604304_sm_miscellaneous_information.pdf

SUPPORTING INFORMATION

Table of Contents

1. Supplementary Methods **3**

1.1 Materials 3

1.2 Bacterial strains 3

1.3 Complementation plasmids 3

1.4 *Streptomyces scabies* fermentation conditions 3

1.5 RT-PCR analysis 4

1.6 LC-MS analysis of extracts 4

1.7 Protein expression 5

1.8 BtmM assays with BtmD 6

1.9 Production of *O*-desmethyl bottromycin 6

1.10 BtmB assays 7

1.11 Isomerisation of **13** 7

1.12 Deuterium labelling of hydrolysed **13** 7

2. Supplementary Tables **8**

Table S1 Primers used in this study 8

Table S2 Plasmids used in this study 9

Table S3 MS data for bottromycin-like metabolites 9

Table S4 Revised bottromycin gene cluster data 10

Table S5 Selected untargeted metabolomic data across all strains 11

3. Supplementary Figures **12**

Figure S1 Bottromycin gene cluster 12

Figure S2 Chromatograms for WT *S. scabies* and complemented mutants 12

Figure S3 RT-PCR analysis of the wild-type and *ΔbtmD* strains 13

Figure S4 Chromatograms for WT and mutant *S. scabies* strains 14

Figure S5 MS network view of WT and mutant *S. scabies* metabolomes 15

Figure S6 LC-MS^2 data for bottromycins **1**, **2**, **3**, **4** and **5** 16

Figure S7 LC-MS^2 data for truncated cyclized metabolite **6** 17

Figure S8 LC-MS^2 data for truncated cyclized metabolites **7**, **8** and **9** 18

Figure S9 LC-MS data for linear metabolites **10** and **11** 19

Figure S10 MS^2 and MS^3 data for **10** and **11** 20

Figure S11 LC-MS^2 data for carboxylated bottromycins **12** and **13** 21
Figure S12 Time course for epimerisation of **13** 22
Figure S13 LC-MS^2 data for hydrolysed **13** 22
Figure S14 LC-MS^2 data for **13** hydrolysed in D_2O 23
Figure S15 LC-MS^n data for **14** 24
Figure S16 LC-MS^n data for **15** and **16** 25
Figure S17 LC-MS^n data for **17** 26
Figure S18 LC-MS^2 data for compounds **18** – **20** 27
Figure S19 LC-MS^n data for **1** indicating distinctive fragments for the macrocycle 28
Figure S20 LC-MS^n data indicating macrocycle fragments for **1**, **4** and **13** 29
Figure S21 LC-MS^n data indicating macrocycle fragments for **2**, **5**, **8** and **12** 30
Figure S22 LC-MS^n data indicating macrocycle fragments for **3**, **6** and **7** 31
Figure S23 Relative proportions of metabolites produced across mutants 32
Figure S24 Time-course analysis of bottromycins produced by wild-type *S. scabies* 33
Figure S25 BtmM activity with BtmD 34
Figure S26 The efficiency of BtmD methionine cleavage by BtmM with various additives 34
Figure S27 LC-MS analysis of *S. scabies ΔbtmJ* + *ΔbtmD* co-culture experiment 35
Figure S28 *In vitro* BtmB activity with **4** 35

4. Supplementary References **36**

1. Supplementary Methods

1.1 Materials

All chemicals were analytical grade and obtained from Sigma Aldrich unless otherwise specified. Antibiotics were used at final concentrations as follows: kanamycin was used at 50 μg mL^{-1}, apramycin at 50 μg mL^{-1}, carbenicillin at 30 μg mL^{-1}, chloramphenicol at 25 μg mL^{-1} and nalidixic acid at 25 μg mL^{-1}.

1.2 Bacterial strains

Spores of *Streptomyces scabies* DSM 41658 were prepared from cultures on instant potato mash agar (20 g L^{-1} Smash (Premier Foods), 20 g L^{-1} agar) and were stored as spore suspensions in 20% glycerol at -20 °C. *E. coli* NovaBlue competent cells (Novagen) were used for genetic manipulation, and the methylation deficient strain of *E. coli,* ET12657 was used[1] for transfer of genetic material to *S. scabies* by conjugation. *E. coli* cells transformed with plasmids were stored in a 30% glycerol stock at -80 °C. The preparation of *S. scabies ΔbtmC*, *ΔbtmD*, *ΔbtmE*, *ΔbtmF*, *ΔbtmG*, *ΔbtmI* and *ΔbtmJ* has been previously described[2].

1.3 Complementation plasmids

Complementation plasmids were generated using the integrative vector pIB139, which contains the strong constitutive *ermE** promoter. For *ΔbtmD* complementation, the *btmD* gene was amplified using primers bottro-rev (EcoRI) and btmD-RBS (AseI), which adds a ribosome binding site (RBS) and an NdeI site to the 5' end of *btmD*. This was digested with AseI/EcoRI and ligated into NdeI/EcoRI-treated pIB139 to generate pIB139-RBS-*btmD*. All other pIB139-RBS vectors were generated from this construct by digestion with NdeI/EcoRI to remove the *btmD* gene and replacing this with *btm* genes amplified using the primers in Table S1. Plasmids were transformed into *E. coli* ET12657 containing the pUZ8002 plasmid, which was then used for conjugation into *S. scabies* as previously described[2].

1.4 *Streptomyces scabies* fermentation conditions

Triplicate cultures were prepared by inoculating 25 mL GYM medium (0.4% glucose, 0.4% yeast extract, 1.0% malt extract, in Milli-Q (MQ) water) with 50 μL of a *S. scabies* spore suspension in a 250 mL flask and incubating at 30 °C, 250 rpm for 48 hours. Seed culture (200 μL) was then used to inoculate 10 mL production medium (PM: 1% glucose, 1.5% starch, 0.5% yeast extract, 1.0% soy flour, 0.5% NaCl, 0.3% $CaCO_3$, 25 μg mL^{-1} $CoCl_2$ in MQ water) in 50 mL conical centrifuge tubes with the caps replaced with foam bungs. These were incubated at 28 °C, 230 rpm for 72 hours. An equal volume of acetonitrile (MeCN) was added to each sample and the mixture was vortexed then centrifuged for 5 minutes at 13,000 rpm. The time course experiment was carried out in triplicate by transferring 1 mL seed culture to 50 mL PM in 250 mL flasks with springs and incubating at 28 °C with shaking at 230 rpm. Samples were taken at 24 hour intervals

during 10 days and stored frozen until their analysis. Co-cultures involved transferring 0.5 mL seed culture from each mutant strain into 50 mL PM and fermenting the culture as described above for 6 days.

1.5 RT-PCR analysis

Both WT *S. scabies* and *ΔbtmD* were grown in bottromycin production medium following the procedure described above. After 72 h, 2 mL culture samples of each strain were collected, washed with an equal volume of RNA*later*® (ThermoFisher Scientific) and cell pellets were stored at -80 °C until processing. RNA was then extracted according to published procedures[3,4]. The mycelium was resuspended in 1 ml of RLT buffer from the RNeasy kit (Qiagen) and then transferred to lysing matrix B tubes (MP Biomedicals) to undergo mechanical lysis using a FastPrep instrument (Bio101). The lysis program consisted of 3 pulses of 30 s at 6.0 m/s with 1 min cooling intervals on ice. After centrifuging the lysed samples for 10 min at 13,000 rpm the supernatants were transferred to fresh tubes and treated following the instructions of the RNeasy Kit. Possible DNA contaminating the samples was removed twice, once with the on-column DNAseI treatment from Qiagen and a second time after elution with TURBO DNA-*free* Kit (Ambion, Invitrogen). The resulting RNA was quantified using a NanoDrop, and approximately 250 ng of each sample were used for cDNA synthesis with the QuantiTect Reverse Transcription Kit (Qiagen), following manufacturer's instructions. The obtained cDNA was used as a template in PCRs with Taq polymerase and specific primers used to assess the expression of relevant genes in the different operons of the *btm* gene cluster (Table S1).

1.6 LC-MS analysis of extracts

Spectra were obtained using a Shimadzu Nexera X2 UHPLC coupled to a Shimadzu IT-TOF mass spectrometer. Samples (5 μL) were injected onto a Phenomenex Kinetex 2.6 μm XB-C18 column (50 mm x 2.1 mm, 100 Å) set at a temperature of 40 °C, eluting with a linear gradient of 5 to 95% acetonitrile in water + 0.1% formic acid (FA) over 6 minutes with a flow-rate of 0.6 mL min^{-1}. Positive mode mass spectrometry data was collected between *m/z* 200 and 2000, and MS^2 data was collected in a data-dependent manner using collision-induced dissociation of the most abundant singly charged species in a scan, with an exclusion time of 0.8 seconds.

Untargeted comparative metabolomics was carried out on triplicate data using Profiling Solution 1.1 (Shimadzu) with an ion m/z tolerance of 100 mDa, a retention time (RT) tolerance of 0.1 min and an ion intensity tolerance of 100,000 units. The data was filtered by removing all species that appeared in either *ΔbtmD* or unmodified production medium. Any species with a p-value >0.002 were omitted and the matrix only displays data from 0.5 – 4 min (Figure S5). Triplicate LC-MS^2 data was used to construct mass spectral networks using the Global Natural Products Social Molecular Networking server (GNPS, http://gnps.ucsd.edu). The following settings were used for analysis: parent mass tolerance = 1 Da, ion tolerance = 0.5 Da, minimum pair cosine = 0.6, minimum matched peaks = 3, minimum cluster size = 2, minimum peak intensity = 25. Network data was visualized and manually cropped in Cytoscape 2.8.3.

Manual cropping consisted of removal of nodes with an RT < 30 s, removal of $[M+Na]^+$ nodes when a corresponding $[M+H]^+$ node is observed, removal of any nodes erroneously duplicated by GNPS, and removal of any nodes that resulted from fewer than 3 spectra. Edges between nodes were sized according to similarity (a linear gradient of 1 to 20 points between cosine 0.6 and 1). The peak areas of extracted ion chromatograms for each node related to bottromycin were quantified in triplicate and imported into Cytoscape (areas were only reported if they appeared in every triplicate sample for a given mutant). The square root of these areas was used to define node diameter ($\sqrt{area}$ = 0, 20 points; $\sqrt{area}$ = maximum (7613), 120 points). A node for 406.27 (identified by Profiling Solution) was manually inputted into the network as an unconnected node.

Additional high-resolution mass spectra were acquired on a Synapt G2-Si mass spectrometer (Waters) operated in positive mode with a scan time of 1.5 s in the mass range of m/z 100-1200. Samples (5 µL) were injected onto an Acquity BEH C18 reversed phase column (1.7 µm, 2.1 x 50 mm, Waters) and eluted with a gradient of 0-90% acetonitrile in water (+ 0.1% formic acid) over a time of 11 min with a flow rate of 0.4 mL/min. Capillary voltage was 0.5 V, cone voltage 30 V, source temperature 100°C, desolvation temperature 250°C. Leu-enkephalin peptide was used to generate a dual lock-mass calibration with *m/z*= 278.1135 and *m/z*=556.2766 measured every 10 s during the run.

1.7 Protein expression

The *btmM* gene was amplified from genomic DNA using primers btmM-start and btmM-end. Following digestion with NdeI and EcoRI, the gene was ligated into NdeI/EcoRI digested pET28(a)+ (Novagen) to generate a construct for the expression of N-terminally His_6-tagged BtmM. pET28-*btmM* was transformed into *E. coli* BL21(DE3) (Novagen), which was were grown in LB medium (1 L) containing kanamycin (50 mg/mL) with shaking at 37 °C until the OD_{600} reached approximately 0.6. Isopropyl-b-D-thiogalactopyranoside (IPTG) was then added to a final concentration of 15 µM and cell growth was continued with shaking at 25 °C for 48 h at 200 rpm. The cells were then harvested by centrifugation and the resulting cell pellet was resuspended in 30 mL binding buffer (10 mM imidazole, 0.5 M NaCl, 20 mM Tris-HCl pH 7.9, 10% glycerol) and lysed by sonication. The resulting cell lysate was clarified by centrifugation and BtmM-His_6 was purified by immobilized nickel-affinity chromatography at 4 °C. The protein eluate was desalted using an Amicon Ultra centrifugal filter (Millipore, 30 kDa MWCO) and the buffer was exchanged to 50 mM Tris-HCl pH 8, 500 mM NaCl. Glycerol was added to 40% and aliquots were frozen in liq. N_2 and stored at -80 °C. Protein identity was confirmed by LC-MS analysis using a Hewlett-Packard HPLC 1100 series instrument coupled to a Thermo Finnigan LCQ ion trap mass spectrometer: BtmM observed mass = 51,360 (predicted mass after loss of N-formyl-methionine = 51,369 Da). BtmB was cloned and expressed in an analogous way to BtmM using primers btmB-start and btmB-end. The procedure was modified by inducing expression using 200 µM IPTG and then shaking at 16 °C for 16 h at 220 rpm. Additionally, a 10 kDa MWCO Amicon filter was used for buffer exchange.

The *btmD* gene was amplified from genomic DNA using primers bottro-for and btmD_end_vector. The btmD_end_vector primer was designed to remove the *btmD* stop codon and introduce sequence to code for a thrombin cleavage site before a HindIII site. Following digestion with NdeI and HindIII, the gene was ligated into NdeI/HindIII digested pET29 (Novagen) to generate a construct for the expression of *C*-terminally His_6-tagged BtmD with the following sequence:

MGPVVVFDCMTADFLNDDPNNAELSALEMEELESWGAWDGEATSLVPRGSKLAAALEHHHHHH.

pET29-*btmD* was transformed into *E. coli* BL21(DE3) (Novagen), which was then grown in LB medium (1 L) containing kanamycin (50 mg/mL) with shaking at 37 °C until the OD_{600} reached approximately 0.6. IPTG was then added to a final concentration of 0.5 mM and cell growth was continued with shaking at 37 °C for 3 h at 250 rpm. The protein was then purified as described for BtmM, but instead used a 3 kDa MWCO Amicon filter. Protein identity was confirmed by LC-MS (Figure S24).

1.8 BtmM assays with BtmD

BtmM was incubated with BtmD using a variety of concentrations and conditions. For a comparison of conditions for activity. 6.4 μM BtmM was tested in 50 mM Tricine pH 9 at 30 °C for 3 hours with the following variables: (i) no added divalent metals, (ii) 32 μM $ZnCl_2$, (iii) 32 μM $CoCl_2$, (iv) 320 μM $ZnCl_2$, (v) 5 mM TCEP and 32 μM $ZnCl_2$. Assays were analyzed using a Thermo Finnigan Surveyor HPLC system coupled to a Thermo Finnigan LCQ Deca ion trap mass spectrometer. Samples were injected onto a Phenomenex Aeris WIDEPORE 3.6 μm C4 column (100 mm x 2.1 mm, 200 Å) set at a temperature of 30 °C, eluting with a linear gradient of 10 to 90% acetonitrile (+ 0.1% trifluoroacetic acid, TFA) in water (+ 0.1% TFA) over 14 minutes with a flow-rate of 0.35 mL min^{-1}.

1.9 Production of *O*-desmethyl bottromycin

The methyl ester of bottromycin was hydrolyzed using LiOH, according to the protocol of Kobayashi *et al.*[5]. THF (4.5 mL) was added to bottromycin A_2 purified from a *S. scabies* culture (175 mL fermentation volume). 500 μL of this was mixed with 500 μL of 1 M LiOH, and the mixture stirred at room temperature for 4 h. This was quenched with 500 μL of 1 M HCl, and the THF was removed by evaporation under reduced pressure. The remaining solution (500 μL) was subjected to purification by preparative HPLC in order to separate hydrolyzed and intact bottromycin, which separate by ~1.5 min in the HPLC conditions (below). The fractions containing the hydrolyzed bottromycin were pooled, freeze-dried and dissolved in H_2O (500 μL). Preparative HPLC was carried out using a Phenomenex Luna 10 μm C18(2) 250 mm x 21.2 mm column connected to an Agilent 1200 series HPLC eluting with a linear gradient of 5 to 95% acetonitrile in water over 40 minutes with a flow-rate of 20 mL min^{-1}.

1.10 BtmB assays

O-desmethyl bottromycin A_2 (25 µL) was mixed with BtmB (5 µM), *S*-adenosyl methionine (SAM) (2 mM) in a final volume of 100 µL in 5 mM Tris-HCl pH 7.5. The full reaction, along with control reactions without either hydrolyzed bottromycin, SAM, or BtmB were incubated at 30 °C for 30 min. The reactions were quenched with an equal volume of methanol, and the samples were analyzed by LC-MS using the same method as described in Crone *et al*.[2]. This LC-MS method provides different ionization parameters and retention times to the other data reported, and is shown in Figure S27.

1.11 Isomerisation of 13 produced by *S. scabies ΔbtmJ*

A 5-day production culture of *S. scabies ΔbtmJ* (400 mL PM) was mixed with MeOH (400 mL) for 20 minutes. The resulting methanolic supernatant was concentrated to 50 mL by rotary evaporation and 50 µL of this solution was purified using a Shimadzu LCMS-2020 eluting with a linear gradient of 5 to 40% acetonitrile in water (+ 0.1% FA) over 20 minutes with a flow-rate of 3.5 mL min^{-1} on a Phenomenex Luna 5µm C18 column (250 x 10 mm, 100 Å). To avoid rapid thiazoline hydrolysis of **13**, fractions were collected in tubes containing Tris-HCl to provide a final concentration of 100 mM Tris-HCl with pH 7.5. This buffered solution was then incubated at room temp. and then analysed on the LCMS-2020 using a Phenomenex Kinetex 2.6 µm XB-C18 column (50 mm x 2.1 mm, 100 Å) eluting with a linear gradient of 5 to 95% acetonitrile in water (+ 0.1% FA) over 6 minutes. This result is shown in Figure S11.

1.12 Deuterium labelling of hydrolysed 13

100 uL of a crude extract of **13** was dried *in vacuo* (GeneVac miVac DUO), resuspended in D_2O (100 uL) and incubated at room temperature for 48 hours. 2 uL of DCl (2M in D_2O) was then added and the acidified sample was incubated at room temperature for 2 hours. This was dried *in vacuo* again, resuspended in H_2O (100 uL) and incubated at room temperature for a further 2 hours to facilitate proton back exchange. An equivalent non-deuterium labelled compound was prepared in H_2O using 0.1% formic acid (hydrolysis occurred spontaneously following HPLC purification containing 0.1% FA when the acid was not neutralised). These samples were then analysed by LC-MS^2 using a Shimadzu IT-TOF as described in section 1.6. The results are shown in Figures S12 and S13.

2. Supplementary Tables

Table S1 Primers used in this study.

Primer Name	Primer Sequence (5'→3')	Restriction site
btmB-start	GAGGCGAGAGGTCATATGAAGATTTCCC	NdeI
btmB-end	CTGGCGCCTCGGAATTCCTGGCG	EcoRI
btmC-start	CGGGGGTTGATCATATGCCTACCGCCA	NdeI
btmC-end	GCGCTCTTCTGAATTCCGGCGCTC	EcoRI
bottro-for	GACCACCATATGGGACCCGTAGTCG	NdeI
bottro-rev	CGGCGAATTCTCATGAGGTGGCTTC	EcoRI
btmE-start	GGGAGAGGGCATATGCGCGAAGCG	NdeI
btmE-end	CTGCCGGAATTCCGTCTCGCGCTC	EcoRI
btmF-start	AGGAGGACATATGACCCGTCCCGC	NdeI
btmF-end	ACAGGGAATTCCAGGGCGGTGCCAC	EcoRI
btmG-start	CGGGGGTGCGCGCATATGAAGCTC	NdeI
btmG-end	ACCACCTCGAAGCTTTCCGTCGCAC	HindIII
btmI-start	ACGGCGGGGGCCATATGACGGCCA	NdeI
btmI-end	GTCCGGTTCCGCGGGGAATTCCGC	EcoRI
btmJ-start	GGCCCGCTGCCATATGGACTTCGAC	NdeI
btmJ-end	GTTTCGGAATTCGCGCCCTCACCAG	EcoRI
btmM-start	GCGTATGACCATATGACCCGGGTCGT	NdeI
btmM-end	CGCGGCGGAATTCCCGGGCCTAC	EcoRI
btmD_end_vector	ATAAAGCTTGGAACCGCGTGGCACCAGTGAGGTGGCT TCACCGTCCCAGGC	HindIII
btmD_RBS	CAGAATTAATAAAGGAGGACCACCATATGGGACCCG TAGTCG	AseI, NdeI
RT_hrdB_fw	GGACCTTGCCGATCTGCTTGA	
RT_hrdB_rv	GGGGAAAGGCTGAGGGGCA	
qRT-A_fw	GATGTCCGGTGCGAGGGCT	
qRT-A_rv	CCGTCGTCTGGTTCGTCGTCC	
qRT-B_fw	CGATCAGCCGCAACCCG	
qRT-B_rv	ACCCGCTCCCTGACCCTCTT	
qRT-C_fw2	GCAACTGCGAGGGTTCCATG	
qRT-C_rv2	GTGGAGGAGGGTGACGAAGG	
qRT-D_fw2	GTATTCGACTGCATGACCGC	
qRT-D_rv2	CCAGGACTCCAGCTCCTCCA	
qRT-E_fw	ACCCGTCTCTGCTTCGCCC	
qRT-E_rv	GGTCCTCCTCACTTCTCCCGT	
qRT-L_fw2	CAGAGGCCAAGGTCAACGC	
qRT-L_rv2	CAGCAGCTCCTCGTCCAC	

Table S2 Plasmids used in this study.

Name	Source	Resistance Marker	Function
pUZ8002	[6]	Kanamycin	Facilitates transfer of genetic material by conjugation
pIB139-RBS-*btmC*	This study	Apramycin	Complementation of *ΔbtmC*
pIB139-RBS-*btmD*	This study	Apramycin	Complementation of *ΔbtmD*
pIB139-RBS-*btmE*	This study	Apramycin	Complementation of *ΔbtmE*
pIB139-RBS-*btmF*	This study	Apramycin	Complementation of *ΔbtmF*
pIB139-RBS-*btmI*	This study	Apramycin	Complementation of *ΔbtmI*
pIB139-RBS-*btmJ*	This study	Apramycin	Complementation of *ΔbtmJ*
pET28-*btmM*	This study	Kanamycin	Expression of *N*-terminally His_6-tagged BtmM
pET29-*btmD*	This study	Kanamycin	Expression of *C*-terminally His_6-tagged BtmD
pET28-*btmB*	This study	Kanamycin	Expression of *N*-terminally His_6-tagged BtmB

Table S3 MS data for bottromycin-like metabolites. All data from wild type *S. scabies* unless otherwise stated.

Compound	Calc. $[M+H]^+$	Shimadzu IT-TOF		Synapt G2-Si	
		Obs. *m/z*	Error (ppm)	Obs. *m/z*	Error (ppm)
**1** (bottromycin A_2)	823.4535	823.4523	-1.46	823.4525	-1.21
**2** (bottromycin B_2)	809.4378	809.4365	-1.61	809.4384	0.74
**3**	795.4222	795.4216	-0.75	795.4221	-0.13
**4** (*O*-desmethyl bottromycin A)	809.4378	809.4377	-0.12	809.4380	0.25
**5** (*O*-desmethyl bottromycin B)	795.4222	795.4196	-3.27	795.4219	-0.38
**6** (from *ΔbtmC*)	452.2867	452.2862	-1.11	452.2864	-0.66
**7**	599.3552	599.3546	-1.00	599.3544	-1.33
**8**	613.3708	613.3686	-3.59	613.3691	-2.77
**9**	627.3865	627.3857	-1.28	627.3845	-3.19
**10** (from *ΔbtmF*)	873.4539	873.4500	-4.47	873.4551	1.37
**11** (from *ΔbtmF*)	887.4695	887.4696	0.11	887.4671	-2.70
**12** (from *ΔbtmJ*)	841.4277	841.4247	-3.57	841.4288	1.31
**13** (from *ΔbtmJ*)	855.4433	855.4411	-2.57	855.4441	0.94
**14**	606.2956	606.2960	0.66	606.2966	1.65
**15**	723.3204	723.3229	3.46	723.3195	-1.24
**16**	737.3361	737.3365	0.54	737.3375	1.90
**17**	406.2699	406.2700	0.25	406.2697	-0.49
**18** (from *ΔbtmC*)	378.2387	378.2381	-1.59	378.2388	0.26
**19** (from *ΔbtmC*)	344.2544	344.2529	-4.36	344.2547	0.87
**20a/b** (from *ΔbtmC*)	330.2387	330.2377	-3.03	330.2391	1.21

Table S4 Revised bottromycin gene cluster data.

**Gene name**	***btm* name**	**Protein family classification**	**Proposed function**
SCAB_56711*	*btmA*	Major facilitator superfamily	Host immunity by export of mature bottromycin
SCAB_56701*	*btmB*	Methyltransferase	*O*-methylation of Asp7
SCAB_56691	*btmC*	Class B radical SAM methyltransferase	β-methylation of Phe6
SCAB_56681	*btmD*	-	Precursor peptide
SCAB_56671	*btmE*	YcaO domain protein	Role in thiazoline formation
SCAB_56661	*btmF*	YcaO domain protein	Role in macrocyclic amidine formation
SCAB_56651	*btmG*	Class B radical SAM methyltransferase	β-methylation of Val4 and Val5
SCAB_56641	*btmH*	α/β hydrolase	Hydrolysis of the follower peptide
SCAB_56631	*btmI*	Metal-dependent hydrolase	Role in macrocyclic amidine formation
SCAB_56621	*btmJ*	Cytochrome P450	Oxidative decarboxylation to convert thiazoline into thiazole
SCAB_56611	*btmK*	Class B radical SAM methyltransferase	β-methylation of Pro2
SCAB_56601	*btmL*	DUF2087	Transcriptional regulator
SCAB_56591	*btmM*	M17 aminopeptidase	Hydrolysis of the *N*-terminal methionine of BtmD

* Genes on opposite strand

Table S5 Selected untargeted metabolomic data across all strains[a].

Compound	RT Range	Ion m/z	Ion RT	PVal	WT1	WT2	WT3	btmC1	btmC2	btmC3	btmD1	btmD2	btmD3	btmE1	btmE2	btmE3	btmF1	btmF2	btmF3	btmG1	btmG2	btmG3	btmI1	btmI2	btmI3	btmJ1	btmJ2	btmJ3	Medium1	Medium2	Medium3
?	003.96 - 003.97	436.282	3.97	4.50E-38	0	0	0	216640	213888	216512	0	0	0	0	0	0	0	0	0	0	0	0	0	0	0	0	0	0	0	0	0
?	002.08 - 002.10	546.056	2.09	1.70E-34	232079	227763	232512	0	0	0	0	0	0	0	0	0	0	0	0	0	0	0	0	0	0	0	0	0	0	0	0
?	001.27 - 001.28	427.190	1.30	5.50E-33	111073	108817	108204	0	0	0	0	0	0	0	0	0	0	0	0	0	0	0	0	0	0	0	0	0	0	0	0
?	002.60 - 002.62	436.229	2.62	1.80E-30	218577	220672	226716	0	0	0	0	0	0	0	0	0	0	0	0	0	0	0	0	0	0	0	0	0	0	0	0
?	003.34 - 003.37	551.381	3.37	1.60E-27	0	0	0	0	0	0	0	0	0	0	0	0	0	0	0	0	0	0	0	0	0	154648	160837	163257	0	0	0
[5+2H]2+	001.96 - 002.01	398.719	1.99	1.80E-27	1691820	1667240	1759418	0	0	0	0	0	0	0	0	0	0	0	0	0	0	0	0	0	0	0	0	0	0	0	0
[2+2H]2+	002.18 - 002.29	405.225	2.26	1.30E-26	20193993	19726090	20981440	0	0	0	0	0	0	0	0	0	0	0	0	0	0	0	0	0	0	0	0	0	0	0	0
[1+2H]2+	002.28 - 002.36	412.233	2.33	2.10E-25	46043334	46765043	49329721	0	0	0	0	0	0	0	0	0	0	0	0	0	0	0	0	0	0	0	0	0	0	0	0
?	002.07 - 002.16	512.073	2.14	3.80E-25	649241	651763	619905	0	0	0	0	0	0	0	0	0	0	0	0	340105	377593	366535	0	0	0	0	0	0	0	0	0
?	003.94 - 004.00	454.295	3.97	4.70E-25	0	0	0	3114783	2982845	3020363	0	0	0	0	0	0	0	0	0	0	0	0	2225904	2206366	2039363	400072	354787	307064	0	0	0
[13+2H]2+	001.93 - 001.99	428.727	1.96	6.50E-25	582649	540352	710443	0	0	0	0	0	0	0	0	0	0	0	0	0	0	0	0	0	0	4322825	4548645	4603083	0	0	0
[3+2H]2+	002.23 - 002.26	398.217	2.24	7.40E-25	1732418	1658558	1793499	0	0	0	0	0	0	0	0	0	0	0	0	0	0	0	0	0	0	0	0	0	0	0	0
[13+2H]2+	002.06 - 002.11	428.225	2.08	1.20E-24	654418	645844	704783	0	0	0	0	0	0	0	0	0	0	0	0	0	0	0	404865	433149	358087	6857087	6963438	7378275	0	0	0
?	003.02 - 003.07	362.304	3.06	1.70E-24	263452	243584	259264	0	0	0	0	0	0	0	0	0	0	0	0	0	0	0	0	0	0	0	0	0	0	0	0
[4+2H]2+	002.04 - 002.09	405.724	2.06	3.20E-24	4808902	4501293	4448707	0	0	0	0	0	0	0	0	0	0	0	0	0	0	0	0	0	0	0	0	0	0	0	0
?	003.83 - 003.91	475.290	3.86	4.00E-24	0	0	0	600833	641644	599524	0	0	0	0	0	0	331787	311056	332366	159457	111104	137672	833276	838348	859535	770104	724259	742073	0	0	0
[7+H]+	001.66 - 001.74	599.345	1.72	7.00E-24	1953356	2073562	1929422	8596046	9272341	9000012	0	0	166577	186594	134468	166891	0	0	0	4161924	4483376	4402285	233682	210933	294333	278354	329965	387768	0	0	0
?	003.17 - 003.20	448.267	3.18	1.50E-23	0	0	0	0	0	0	0	0	0	0	0	0	0	0	0	198760	184337	183094	0	0	0	0	0	0	0	0	0
?	003.47 - 003.52	285.243	3.48	2.20E-23	0	0	0	787388	740285	769920	0	0	0	0	0	0	0	0	0	0	0	0	623808	680841	611081	0	0	0	0	0	0
Source MS fragment of 1?	002.26 - 002.27	322.214	2.27	5.50E-23	150969	158311	143325	0	0	0	0	0	0	0	0	0	0	0	0	0	0	0	0	0	0	0	0	0	0	0	0
?	002.69 - 002.72	226.214	2.71	7.10E-23	265216	265288	242752	0	0	0	0	0	0	0	0	0	0	0	0	0	0	0	0	0	0	0	0	0	0	0	0
Source MS fragment of 13?	001.94 - 001.99	380.127	1.97	1.70E-22	0	0	0	0	0	0	0	0	0	0	0	0	0	0	0	0	0	0	0	0	0	285376	286281	260413	0	0	0
[11+H]+	002.19 - 002.25	887.471	2.21	2.60E-22	0	0	0	0	0	0	0	0	0	0	0	0	597863	588450	645701	0	0	0	520451	469438	494260	0	0	0	0	0	0
?	001.61 - 001.64	475.952	1.63	4.40E-22	0	0	0	0	0	0	0	0	0	393600	377961	421760	0	0	0	0	0	0	0	0	0	0	0	0	0	0	0
[4+2H]2+	002.03 - 002.10	405.224	2.06	7.40E-22	8333223	8893546	9348495	0	0	0	0	0	0	0	0	0	0	0	0	0	0	0	0	0	0	0	0	0	0	0	0
[14+H]+	001.76 - 001.82	606.296	1.79	8.30E-22	1379358	1503694	1685650	0	0	0	0	0	0	0	0	0	4158355	4088050	4419473	0	0	0	1846729	1872286	1800853	751572	699748	851694	0	0	0
?	003.67 - 003.75	440.277	3.70	1.40E-21	0	0	0	4893525	4466142	4872521	0	0	0	0	0	0	0	0	0	0	0	0	4174024	3802042	4204354	602424	533332	408961	0	0	0
?	003.07 - 003.10	257.618	3.09	1.50E-21	0	0	0	0	0	0	0	0	0	205995	197482	182912	0	0	0	0	0	0	0	0	0	0	0	0	0	0	0
[5+2H]2+	001.96 - 002.02	398.216	1.99	1.70E-21	2974505	3343775	3247405	0	0	0	0	0	0	0	0	0	0	0	0	0	0	0	0	0	0	0	0	0	0	0	0
?	000.71 - 000.74	486.318	0.73	3.60E-21	0	0	0	146045	142806	129547	0	0	0	0	0	0	0	0	0	0	0	0	0	0	0	0	0	0	0	0	0
?	003.67 - 003.75	299.259	3.70	6.50E-21	0	0	0	3250286	3072183	3410034	0	0	0	0	0	0	0	0	0	0	0	0	2670854	2315968	2488586	348588	331520	356674	0	0	0
?	002.50 - 002.55	228.195	2.52	7.40E-21	3438598	3288848	3716211	258496	144448	249920	0	0	0	0	0	0	0	0	0	0	0	0	0	0	0	0	0	0	0	0	0
?	001.71 - 001.74	572.333	1.78	1.00E-20	0	0	0	0	0	0	0	0	0	0	0	0	0	0	0	0	0	0	0	0	0	113672	121838	129844	0	0	0
?	001.70 - 001.73	602.367	1.70	1.10E-20	0	0	0	180951	159605	177949	0	0	0	0	0	0	0	0	0	0	0	0	0	0	0	0	0	0	0	0	0
[17+H]+	001.57 - 001.64	406.270	1.61	1.40E-20	5113933	4890936	4909572	0	0	0	0	0	0	1967875	2011261	1762592	4375755	3772846	4292633	0	0	0	2555874	2770577	2653191	3312425	3380896	3527367	0	0	0
?	002.75 - 002.76	398.340	2.77	1.50E-20	0	0	0	0	0	0	0	0	0	0	0	0	0	0	0	0	0	0	124224	141120	127744	0	0	0	0	0	0
[12+2H]2+	001.97 - 002.04	421.218	2.02	1.90E-20	1759689	1945285	1920678	0	0	0	0	0	0	0	0	0	0	0	0	0	0	0	0	0	0	1257259	1476333	1403513	0	0	0
?	002.30 - 002.32	250.154	2.32	2.40E-20	306250	347625	311785	0	0	0	0	0	0	0	0	0	0	0	0	0	0	0	0	0	0	0	0	0	0	0	0
?	001.72 - 001.78	540.374	1.75	3.30E-20	0	0	237491	3013310	2976728	3372744	0	0	0	0	0	0	1811078	1761874	1802280	812919	995192	865471	4356620	4751074	4587155	3038229	3321032	3236413	0	0	0
Source MS fragment of 1?	002.29 - 002.35	476.325	2.34	6.30E-20	2133031	2144508	2423181	0	0	0	0	0	0	0	0	0	0	0	0	0	0	0	0	0	0	0	0	0	0	0	0
?	001.72 - 001.77	425.312	1.75	6.70E-20	0	0	0	1252381	1224706	1240422	0	0	0	0	0	0	523106	636714	650721	267565	272396	269851	2012344	1879616	2005985	1472021	1315514	1220981	0	0	0
?	001.87 - 001.90	498.056	1.89	6.90E-20	257489	291471	295293	0	0	0	0	0	0	0	0	0	0	0	0	0	0	0	0	0	0	0	0	0	0	0	0
?	001.77 - 001.80	509.795	1.79	8.10E-20	0	0	0	155246	165178	179984	0	0	0	0	0	0	0	0	0	0	0	0	0	0	0	0	0	0	0	0	0
[Bottro C+2H]2+	002.39 - 002.44	419.740	2.41	1.10E-19	852685	974188	976581	0	0	0	0	0	0	0	0	0	0	0	0	0	0	0	0	0	0	0	0	0	0	0	0
[Bottro C+2H]2+	002.39 - 002.44	419.240	2.41	1.30E-19	1847306	1671321	1946862	0	0	0	0	0	0	0	0	0	0	0	0	0	0	0	0	0	0	0	0	0	0	0	0
[2+2H]2+	002.22 - 002.29	405.726	2.27	1.70E-19	12433168	11379711	13302028	0	0	0	0	0	0	0	0	0	0	0	0	0	0	0	0	0	0	0	0	0	0	0	0
13+Me?	002.14 - 002.16	435.736	2.15	2.30E-19	0	0	0	0	0	0	0	0	0	0	0	0	0	0	0	0	0	0	0	0	0	163776	182848	157312	0	0	0
?	003.90 - 003.96	428.374	3.92	2.50E-19	0	0	0	1421901	1268275	1224383	0	0	0	0	0	0	0	0	0	0	0	0	0	0	0	0	0	0	0	0	0
?	001.75 - 001.81	268.187	1.77	4.70E-19	285075	275808	321624	0	0	0	0	0	0	0	0	0	0	0	0	0	0	0	0	0	0	0	0	0	0	0	0
?	003.83 - 003.91	299.257	3.89	5.70E-19	147712	243181	146944	1201516	1197169	1260207	0	0	0	0	0	0	724810	568300	598235	192549	196270	170816	1700126	1616500	1637062	1280789	1481209	1498897	0	0	0
?	003.67 - 003.80	452.279	3.78	6.20E-19	0	0	0	911046	937208	896334	0	0	0	0	0	0	0	0	0	0	0	0	497939	502117	364352	0	0	0	0	0	0
?	003.94 - 004.00	313.274	3.97	6.80E-19	0	0	0	1869183	1793555	1662129	0	0	0	0	0	0	0	0	0	0	0	0	1223691	1231660	1017797	166912	137344	219648	0	0	0
?	002.25 - 002.27	264.133	2.26	9.30E-19	241452	276736	237650	0	0	0	0	0	0	0	0	0	0	0	0	0	0	0	0	0	0	0	0	0	0	0	0
[13+2H]2+	001.93 - 001.99	428.225	1.96	1.40E-18	946606	1174805	980796	0	0	0	0	0	0	0	0	0	0	0	0	0	0	0	0	0	198481	7723451	8101721	9082398	0	0	0
?	002.03 - 002.04	606.322	2.04	1.40E-18	0	0	0	168563	157626	187279	0	0	0	0	0	0	0	0	0	0	0	0	0	0	0	0	0	0	0	0	0
?	003.83 - 003.88	672.383	3.87	1.40E-18	0	0	0	189630	231074	206391	0	0	0	0	0	0	0	0	0	0	0	0	269798	240487	245714	200033	184601	210497	0	0	0
[9+H]+	001.95 - 002.01	627.375	1.96	2.20E-18	883139	914169	951723	0	0	0	0	0	0	0	0	0	220851	220870	253835	0	0	0	0	0	0	0	124288	0	0	0	0
?	001.19 - 001.19	552.790	1.20	2.60E-18	0	0	0	125882	128768	108480	0	0	0	0	0	0	0	0	0	0	0	0	0	0	0	0	0	0	0	0	0
?	001.10 - 001.15	499.323	1.11	2.70E-18	171679	148032	175882	0	0	0	0	0	0	0	0	0	0	0	0	0	0	0	0	0	0	0	0	0	0	0	0
[13+H]+	002.05 - 002.11	855.445	2.08	2.70E-18	0	0	177048	0	0	0	0	0	0	0	0	0	0	0	0	0	0	0	142303	0	187903	2402351	2659894	2335216	0	0	0
[11+2H]2+	002.19 - 002.25	444.237	2.22	4.60E-18	0	0	0	0	0	0	0	0	0	0	0	0	1546534	1350272	1551301	0	0	0	1372603	1415704	1215246	0	133888	0	0	0	0
[11+Na]+	002.20 - 002.22	909.453	2.22	5.20E-18	0	0	0	0	0	0	0	0	0	0	0	0	223814	245841	222238	0	0	0	231431	221778	185366	0	0	0	0	0	0
?	003.29 - 003.37	401.338	3.33	5.70E-18	396254	286080	378433	1443785	1430370	1563330	0	0	0	0	0	0	763764	775857	795032	507350	393664	486435	495861	468572	437014	1093562	1219847	1285170	0	0	0
?	003.78 - 003.84	402.358	3.81	5.70E-18	0	0	0	2696760	3155863	2932547	0	164928	180480	0	0	0	0	0	121024	0	0	0	0	0	0	0	0	0	140352	163584	0
[1+2H]2+	002.28 - 002.36	412.734	2.34	5.90E-18	27125669	29809169	32798387	0	0	0	0	0	0	0	0	0	0	0	0	0	0	0	0	0	0	0	0	0	0	0	0
[13+2H]2+	002.04 - 002.11	428.727	2.08	7.20E-18	260099	371520	298792	0	0	0	0	0	0	0	0	0	0	0	0	0	0	0	160256	0	219968	3239039	3370712	3819149	0	0	0
Source MS fragment of 1?	002.30 - 002.36	278.150	2.33	1.20E-17	755703	620972	678071	0	0	0	0	0	0	0	0	0	0	0	0	0	0	0	0	0	0	0	0	0	0	0	0
[14+2H]2+	001.77 - 001.82	303.650	1.80	1.60E-17	986772	921125	793250	0	0	0	0	0	0	0	0	0	2151521	2426348	2565575	0	0	0	736969	750249	777138	433542	369292	338240	0	0	0
?	003.46 - 003.53	426.262	3.49	1.60E-17	0	0	0	1261964	1104865	1294579	0	0	0	0	0	0	0	0	0	0	0	0	1064395	987930	855774	0	0	0	0	0	0
?	002.76 - 002.79	436.230	2.77	1.80E-17	527861	437678	455901	238882	245300	274615	0	0	0	0	0	0	0	0	0	0	0	0	0	0	0	0	0	0	0	0	0
?	002.00 - 002.02	212.164	2.01	2.10E-17	376847	312473	372850	0	0	0	0	0	0	0	0	0	0	0	0	0	0	0	0	0	0	0	0	0	0	0	0
Source MS fragment of 2?	002.24 - 002.28	377.219	2.27	2.70E-17	405029	329369	364756	0	0	0	0	0	0	0	0	0	0	0	0	0	0	0	0	0	0	0	0	0	0	0	0
?	002.57 - 002.58	468.265	2.57	2.80E-17	0	0	0	0	0	0	0	0	0	0	0	0	0	0	0	0	0	0	0	0	0	110848	117120	134972	0	0	0
?	001.73 - 001.79	624.303	1.77	2.90E-17	0	0	0	0	0	0	0	0	0	0	0	0	246225	305743	280816	0	0	0	137107	127537	130869	0	0	0	0	0	0
Tail of [1+2H]2+	002.53 - 002.59	412.233	2.57	3.70E-17	170368	141568	143936	0	0	0	0	0	0	0	0	0	0	0	0	0	0	0	0	0	0	0	0	0	0	0	0
?	003.79 - 003.81	283.264	3.81	3.70E-17	0	0	0	277819	341760	324416	0	0	0	0	0	0	0	0	0	0	0	0	0	0	0	0	0	0	0	0	0
?	001.41 - 001.42	387.234	1.41	4.90E-17	0	0	0	0	0	0	0	0	0	0	0	0	0	0	0	0	0	0	0	0	0	132140	143966	163141	0	0	0
?	001.86 - 001.95	489.306	1.90	6.20E-17	0	0	0	611604	619789	601597	0	0	0	0	0	0	421495	456420	321268	458032	480800	528279	415406	459839	452945	617576	552554	586298	0	0	0
[7+Na]+	001.93 - 001.99	621.328	1.97	6.20E-17	0	0	0	205081	208231	170277	0	0	0	0	0	0	0	0	0	0	0	0	0	0	0	0	0	0	0	0	0
?	001.84 - 001.87	565.309	1.85	1.20E-16	232912	187904	216003	0	0	0	0	0	0	0	0	0	271332	220992	256949	0	0	0	0	0	0	0	0	0	0	0	0
?	000.85 - 000.94	470.325	0.87	1.30E-16	0	0	0	196501	173890	220224	0	0	0	0	0	0	0	0	0	0	0	0	0	0	0	203765	192829	170101	0	0	0
?	003.68 - 003.74	422.268	3.70	1.40E-16	0	0	0	450441	354462	463431	0	0	0	0	0	0	0	0	0	0	0	0	372454	367838	380295	0	0	0	0	0	0
[14+K]+	001.79 - 001.80	644.258	1.80	1.80E-16	0	0	0	0	0	0	0	0	0	0	0	0	145772	155584	123805	0	0	0	0	0	0	0	0	0	0	0	0
?	001.55 - 001.60	502.270	1.61	2.30E-16	0	0	0	170508	196875	145943	0	0	0	0	0	0	0	0	0	0	0	0	0	0	0	165437	179504	177523	0	0	0
?	001.75 - 001.78	665.333	1.76	2.70E-16	0	0	0	0	0	0	0	0	0	0	0	0	185393	196782	209843	0	0	0	0	0	0	220957	269186	210290	0	0	0
[19+H]+	001.24 - 001.50	344.252	1.41	3.20E-16	192842	182863	278604	1626285	1784872	1832023	0	0	0	186202	217895	213688	528230	559424	589066	718177	660136	818581	1297284	1161716	1202800	937643	1097045	1221078	0	0	0
?	001.68 - 001.70	579.977	1.70	3.80E-16	0	0	0	0	0	0	0	0	0	0	0	0	0	0	0	0	0	0	245952	276441	298752	183296	226870	177247	0	0	0
[10+2H]+	002.10 - 002.15	873.452	2.13	6.50E-16	0	0	0	0	0	0	0	0	0	0	0	0	449147	576062	517434	0	0	0	0	0	0	0	0	0	0	0	0
[10+2H]2+	002.10 - 002.16	437.230	2.13	7.20E-16	305245	232604	311296	0	0	0	0	0	0	0	0	0	1041439	1299477	1119598	0	0	0	457553	454152	387008	0	0	0	0	0	0
18 + Me?	001.64 - 001.85	392.251	1.75	7.40E-16	0	0	0	500556	446453	467496	0	0	0	0	0	0	180299	204928	185041	176173	232802	245187	428472	429552	427094	330053	444446	402218	0	0	0
Source MS fragment of 17?	001.58 - 001.63	241.190	1.61	7.90E-16	1259857	1125042	1208942	0	0	0	0	0	0	346215	324065	409900	1060820	944512	992911	0	0	0	400512	505792	459456	799088	551552	757582	0	0	0
?	002.68 - 002.71	587.189	2.69	8.40E-16	398829	393348	465691	481240	462565	514663	0	0	0	0	0	0	622791	526030	572619	224721	218186	224360	429763	420842	344440	469560	506397	448017	0	0	0
19+Me?	001.50 - 001.63	358.269	1.61	8.70E-16	0	0	0	840878	925250	978372	0	0	0	0	0	0	184392	209698	181416	327215	398832	296064	586122	545724	627389	381086	468838	531038	0	0	0
Source MS fragment of 1?	002.30 - 002.35	363.243	2.33	9.20E-16	624959	776742	791246	0	0	0	0	0	0	0	0	0	0	0	0	0	0	0	0	0	0	0	0	0	0	0	0
?	003.98 - 004.00	381.984	4.00	1.10E-15	150592	184256	192960	0	0	0	0	0	0	0	0	0	0	0	0	0	0	0	0	0	0	0	0	0	0	0	0
?	002.04 - 002.06	641.382	2.06	1.10E-15	123670	152699	123005	0	0	0	0	0	0	0	0	0	0	0	0	0	0	0	0	0	0	0	0	0	0	0	0
?	001.99 - 002.01	562.850	2.00	1.30E-15	0	0	0	259895	318848	254572	0	0	0	0	0	0	0	0	0	0	0	0	0	0	0	0	0	0	0	0	0
[13+Na]+	002.06 - 002.10	877.422	2.08	1.40E-15	0	0	0	0	0	0	0	0	0	0	0	0	0	0	0	0	0	0	0	0	0	314646	260372	248098	0	0	0
?	003.87 - 003.92	372.345	3.89	1.50E-15	0	0	0	743632	651701	678930	0	0	0	0	0	0	0	0	0	343768	399402	239296	0	0	0	0	0	0	0	0	0
?	002.07 - 002.12	721.417	2.10	1.60E-15	0	0	0	819782	897570	931612	0	0	0	0	0	0	326131	338790	413291	213248	199990	280252	182074	199360	279910	471447	499081	590869	0	0	0
?	001.47 - 001.53	454.208	1.51	1.70E-15	0	0	0	171517	131968	151307	0	0	0	0	0	0	0	0	0	0	0	0	0	0	0	0	0	0	0	0	0
?	003.27 - 003.29	392.293	3.28	1.70E-15	0	0	0	0	0	0	0	0	0	0	0	0	0	0	0	0	0	0	0	0	0	140608	147423	178597	0	0	0
?	003.61 - 003.64	482.396	3.63	1.70E-15	0	0	0	0	0	0	0	0	0	0	0	0	0	0	0	176587	230650	172713	0	0	0	218624	210438	240896	0	0	0
?	003.93 - 004.00	466.295	3.97	2.00E-15	0	0	0	401420	497047	481388	0	0	0	0	0	0	0	0	0	0	0	0	259851	320216	238667	0	0	0	0	0	0
Source MS fragment of 1?	002.31 - 002.33	313.104	2.32	2.20E-15	376798	288697	332000	0	0	0	0	0	0	0	0	0	0	0	0	0	0	0	0	0	0	0	0	0	0	0	0
?	003.39 - 003.43	330.263	3.40	2.40E-15	355309	269504	259072	0	0	0	0	0	0	0	0	0	0	0	0	0	0	0	433281	504783	524552	0	0	0	0	0	0
[16+2H]2+	002.09 - 002.14	369.172	2.11	2.60E-15	1675807	1430807	1532422	0	0	0	0	0	0	0	0	0	817021	566080	768063	0	0	0	0	0	130880	150656	0	0	0	0	0
[15+2H]2+	001.96 - 002.02	362.164	1.98	2.80E-15	752289	817967	741096	0	0	0	0	0	0	0	0	0	258816	175488	160512	0	0	0	0	0	0	0	0	134208	0	0	0
[14+2H]2+	001.77 - 001.82	304.153	1.79	2.90E-15	419968	286024	399536	0	0	0	0	0	0	0	0	0	1105081	955011	1127409	0	0	0	512545	415590	363649	192211	164602	127616	0	0	0
?	001.48 - 001.70	443.274	1.54	3.40E-15	328537	343920	300980	564677	590834	554180	0	0	130337	0	0	0	352931	320927	378226	0	0	0	557324	527049	512444	423271	501276	429998	0	0	0
[6+H]+	000.96 - 001.13	452.283	1.09	3.50E-15	0	0	177476	1192072	1247816	1454656	0	0	0	0	0	0	0	0	0	300571	344456	367226	0	0	0	0	0	0	0	0	0
?	001.67 - 001.73	538.359	1.70	4.60E-15	0	0	0	352245	362872	347812	0	0	0	0	0	0	322413	264070	283684	178063	161984	114240	563829	457238	423769	527675	598014	556096	0	0	0
?	003.80 - 003.85	601.374	3.81	5.20E-15	253381	282381	222464	0	0	0	0	0	0	0	0	0	148699	148096	127552	117056	161094	149208	0	0	0	197683	176384	173376	0	0	0
[3+2H]2+	002.15 - 002.25	398.718	2.24	6.00E-15	928126	845504	699968	0	0	0	0	0	0	0	0	0	0	0	0	0	0	0	0	0	0	0	0	0	0	0	0
[2+H+K]2+	002.25 - 002.27	424.191	2.27	6.20E-15	245575	325452	299250	0	0	0	0	0	0	0	0	0	0	0	0	0	0	0	0	0	0	0	0	0	0	0	0
?	002.94 - 002.96	327.066	2.95	8.50E-15	257567	282880	339411	0	0	0	0	0	0	0	0	0	0	0	0	0	0	0	0	0	0	0	0	0	0	0	0
[13+H]+	001.94 - 002.00	855.442	1.96	9.40E-15	0	251266	289272	0	0	0	0	0	0	0	0	0	0	0	0	0	0	0	0	0	0	1993520	2537909	2451411	0	0	0
?	001.74 - 001.81	398.148	1.78	9.70E-15	1442677	1677468	1579380	624398	605335	752336	0	0	0	0	0	139987	544958	692469	574594	523281	459923	560508	566578	530906	587340	504803	541053	671747	0	0	0
?	000.70 - 000.95	295.163	0.88	1.00E-14	1455255	1416117	1532180	916053	936786	876529	0	0	0	1506033	1595793	1602944	842300	720578	741046	944300	997687	818292	646715	675311	871963	904557	953267	954205	0	225011	0
?	001.74 - 001.83	228.195	1.77	1.00E-14	813547	1004057	1016733	282112	332480	331776	0	0	124096	0	0	0	0	0	0	0	0	0	0	0	0	0	0	0	0	0	0
[15+2H]2+	001.97 - 002.00	362.666	1.99	1.30E-14	430016	364726	321664	0	0	0	0	0	0	0	0	0	0	0	0	0	0	0	0	0	0	0	0	0	0	0	0
?	002.39 - 002.67	537.303	2.49	1.30E-14	253415	342487	278792	828818	763914	832516	0	0	0	0	0	0	607261	490096	619548	579765	575658	566206	333642	464240	430947	591810	480168	488336	0	0	0
?	001.76 - 001.89	457.337	1.82	1.30E-14	0	0	0	506300	536973	551513	0	0	0	0	0	0	160384	109312	162085	347042	293314	361797	306731	370352	343487	360144	437858	305502	0	0	0
[14+Na]+	001.74 - 001.83	628.279	1.80	1.40E-14	376411	399618	288311	0	0	0	0	0	0	0	0	0	916311	903379	793109	0	0	0	463702	494372	559217	392015	220045	252212	0	0	0
?	002.66 - 002.72	571.216	2.69	2.60E-14	1851045	1863900	1869880	1974908	1682901	2267772	0	0	0	0	0	0	2397531	2456589	2395119	1380634	1696625	1348348	2217169	2190207	1894771	2549049	2178282	2036407	0	0	0
16+Me?	001.77 - 001.83	620.306	1.80	2.80E-14	0	0	0	0	0	0	0	0	0	0	0	0	587532	555358	537839	0	0	0	199749	128995	173312	127010	0	0	0	0	0
?	002.60 - 002.64	464.263	2.62	4.20E-14	406746	433314	401705	254744	287166	283281	0	0	0	127039	146714	0	599244	524896	612955	325271	288259	298359	303767	256716	364323	780471	714187	714441	0	0	0
?	003.84 - 003.89	637.345	3.88	4.90E-14	0	0	0	169092	232351	173644	0	0	0	0	0	0	0	0	0	0	0	0	208864	237994	242503	239384	180544	227602	0	0	0
Source MS fragment of 1?	002.24 - 002.36	348.136	2.33	5.10E-14	2127936	2048237	2713406	0	0	0	0	0	0	0	0	0	0	0	0	0	0	0	0	0	0	0	0	0	0	0	0
?	003.47 - 003.55	430.201	3.54	5.60E-14	176896	129251	147520	0	0	0	0	0	0	0	0	0	0	0	0	0	0	0	0	0	0	0	0	0	0	0	0
?	000.76 - 001.53	213.158	1.29	7.90E-14	0	0	0	586051	613314	715037	0	0	0	0	0	0	372393	308051	329829	283294	361565	323021	400201	493261	530610	433007	555942	500570	0	0	0
Source MS fragment of 2?	002.23 - 002.29	462.304	2.27	8.00E-14	1028974	832809	1156898	0	0	0	0	0	0	0	0	0	0	0	0	0	0	0	0	0	0	0	0	0	0	0	0
?	002.04 - 002.06	435.234	2.12	8.60E-14	0	0	0	0	0	0	0	0	0	0	0	0	0	0	0	0	0	0	167168	192305	204331	364480	258965	323189	0	0	0
Source MS fragment of 1?	002.25 - 002.35	471.203	2.34	8.70E-14	856304	810961	621823	0	0	0	0	0	0	0	0	0	0	0	0	0	0	0	0	0	0	0	0	0	0	0	0
?	002.71 - 002.75	328.067	2.73	8.70E-14	163712	223856	216576	0	0	0	0	0	0	0	0	0	0	0	0	0	0	0	0	0	0	0	0	0	0	0	0
O-demethyl bottro C?	002.14 - 002.15	412.229	2.14	8.80E-14	210432	154048	169984	0	0	0	0	0	0	0	0	0	0	0	0	0	0	0	0	0	0	0	0	0	0	0	0
Source MS fragment of 14?	001.77 - 001.81	380.127	1.80	9.60E-14	186368	240940	229712	0	0	0	0	0	0	0	0	0	643204	753323	731386	0	0	0	242368	241296	353677	127680	0	0	0	0	0
?	002.12 - 002.15	728.417	2.14	1.00E-13	0	0	0	0	0	0	0	0	0	0	0	0	0	0	0	0	0	0	0	0	0	279047	328217	388071	0	0	0
?	001.48 - 001.53	612.826	1.51	1.10E-13	0	0	0	278181	249856	309952	0	0	0	0	0	0	332821	416276	364039	231847	166720	282139	344337	346468	401277	624404	802141	731634	0	0	0
?	001.17 - 001.27	536.046	1.25	1.20E-13	1011187	855896	821135	195254	154176	0	0	0	0	0	0	0	0	0	0	300676	353677	277496	0	0	0	0	0	0	0	0	0
?	002.04 - 002.08	476.323	2.05	1.20E-13	301527	338969	385922	0	0	0	0	0	0	0	0	0	0	0	0	0	0	0	0	0	0	295516	211430	310145	0	0	0
[11+H]+ isotope	002.20 - 002.23	888.470	2.22	1.30E-13	0	0	0	0	0	0	0	0	0	0	0	0	359512	291703	380243	0	0	0	303708	264048	374277	0	0	0	0	0	0
?	000.92 - 001.10	302.206	1.04	1.40E-13	207304	148803	190080	648928	777590	846332	0	0	0	154348	140327	146688	468084	368384	441312	371679	355600	439616	556654	529696	639051	739141	660345	748977	0	0	0
+Na for 540.37	001.70 - 001.79	562.353	1.75	1.40E-13	0	0	0	459029	405169	498887	0	0	0	0	0	0	262801	310185	305383	414095	306581	329511	647589	735338	561532	507018	424612	427104	0	0	0
?	001.62 - 001.74	487.293	1.70	1.50E-13	292167	369094	294844	343804	301544	370748	0	0	0	238315	287096	299595	309863	302714	310100	447277	398767	434145	345697	379803	325576	451526	386373	426891	0	0	0
?	002.39 - 002.41	252.193	2.40	2.30E-13	166848	220096	163840	0	0	0	0	0	0	0	0	0	0	0	0	0	0	0	0	0	0	0	0	0	0	0	0
?	001.62 - 001.83	477.265	1.65	2.70E-13	0	0	0	458421	392078	426172	0	0	0	0	118865	0	191956	180051	190305	171618	169947	176069	451979	373884	443335	349766	310769	383075	0	0	0
?	003.95 - 003.98	311.259	3.97	2.80E-13	0	0	0	275392	308939	308608	0	0	0	0	0	0	160192	193152	196864	0	0	0	252800	242048	321216	454921	434687	328320	0	0	0
?	000.77 - 001.00	227.174	0.86	3.10E-13	0	0	0	563088	533440	691185	0	0	0	0	0	0	280526	306240	265600	225408	183232	243776	511717	454801	506249	424001	387136	543989	0	0	0
?	000.70 - 000.83	614.272	0.78	4.20E-13	731715	775237	724806	684885	650490	720958	0	0	113920	448217	414698	440269	440893	475220	442188	512417	635068	550111	477829	473479	617323	449327	379326	393718	0	0	0
[16+2H]2+	002.10 - 002.13	369.674	2.11	4.30E-13	891763	693760	684875	0	0	0	0	0	0	0	0	0	236288	378880	268480	0	0	0	0	0	0	0	0	0	0	0	0
?	002.29 - 002.32	493.746	2.32	4.40E-13	0	0	0	0	0	0	0	0	0	0	0	0	0	0	0	0	0	0	387200	305298	275776	0	0	0	0	0	0
[12+2H]2+	001.99 - 002.04	421.718	2.01	5.00E-13	1101680	763264	1081414	0	0	0	0	0	0	0	0	0	0	0	0	0	0	0	0	0	0	687868	666368	815931	0	0	0
?	001.44 - 001.46	517.780	1.46	5.60E-13	206272	203520	146688	0	0	0	0	0	0	0	0	0	0	0	0	0	0	0	0	0	0	0	0	0	0	0	0
?	001.62 - 001.80	475.286	1.70	6.00E-13	132475	0	163463	1034804	1344685	1294615	0	0	0	0	0	0	290372	289064	360963	375115	404275	397544	638998	498049	460832	514770	547530	592735	0	0	0
?	002.49 - 002.54	478.297	2.52	6.10E-13	201927	189691	304769	357329	262968	336268	0	0	0	0	0	0	492439	461712	369024	180420	163406	163840	1001903	908116	1190774	441936	412276	480173	0	0	0
?	001.00 - 001.18	243.132	1.12	6.20E-13	320193	354726	302984	238837	253814	318467	0	0	0	0	0	0	432940	362426	347366	226611	199411	213632	301311	219584	289796	392678	375491	450447	0	0	0
?	002.67 - 002.69	804.437	2.69	6.30E-13	0	0	0	0	0	0	0	0	0	0	0	0	160638	184768	127447	0	0	0	0	0	0	0	0	0	0	0	0
[8+H]+	001.82 - 001.88	613.366	1.84	6.80E-13	844255	1097787	857662	210385	148480	172198	0	0	0	0	0	0	0	0	0	129819	0	0	0	0	0	160357	183315	224301	0	0	0
?	003.08 - 003.09	298.632	3.08	6.90E-13	0	0	0	0	0	0	0	0	0	202496	199232	143208	0	0	0	0	0	0	0	0	0	0	0	0	0	0	0
?	003.19 - 003.20	518.275	3.20	7.00E-13	217335	162475	218674	211886	291859	222848	0	0	0	0	0	0	280861	264346	273439	0	0	0	225865	276748	199800	292903	242906	275434	0	0	0
?	001.63 - 001.69	555.962	1.66	7.50E-13	0	0	0	302417	279552	253248	0	0	0	0	0	0	163392	299840	206848	0	0	0	276587	216512	276246	463988	413246	515800	0	0	0
?	001.28 - 001.32	277.153	1.28	1.10E-12	0	0	0	0	0	0	0	0	0	0	0	0	0	0	0	0	0	0	0	0	0	162834	111040	137344	0	0	0
?	003.19 - 003.24	376.322	3.22	1.20E-12	226240	332480	295680	0	0	0	0	0	0	0	0	0	0	0	0	0	0	0	0	0	0	0	0	0	0	0	0
?	001.30 - 001.33	669.390	1.32	1.20E-12	0	0	0	0	0	0	0	0	0	0	0	0	219990	284041	296840	0	0	0	0	0	0	338458	475877	480509	0	0	0
?	001.50 - 001.67	227.174	1.63	1.20E-12	0	0	0	450717	471679	565210	0	0	0	0	0	0	0	0	130560	176320	176576	170560	339879	318592	357938	282679	300938	361660	0	0	0
[13+Na]+	001.95 - 001.99	877.425	1.96	1.20E-12	0	0	0	0	0	0	0	0	0	0	0	0	0	0	0	0	0	0	0	0	0	355306	242093	299040	0	0	0
?	002.93 - 002.97	326.073	2.95	1.30E-12	495692	337128	448287	0	0	0	0	0	0	0	0	0	0	0	0	0	0	0	0	0	0	0	0	0	0	0	0
Source MS fragment of 1?	002.24 - 002.35	461.222	2.34	1.50E-12	708464	509168	740919	0	0	0	0	0	0	0	0	0	0	0	0	0	0	0	0	0	0	0	0	0	0	0	0
?	000.50 - 000.62	243.133	0.55	1.50E-12	322435	381701	311278	513279	568759	682641	0	0	0	0	0	0	341632	408192	443960	325734	348196	316890	442314	379500	484737	416059	475177	512835	0	0	0
[11+2H]2+	002.20 - 002.23	444.741	2.21	1.50E-12	0	0	0	0	0	0	0	0	0	0	0	0	680384	602880	811876	0	0	0	646026	858927	588605	0	0	0	0	0	0
?	001.06 - 001.14	536.046	1.10	1.80E-12	228962	243520	324728	0	0	0	0	0	0	0	0	0	0	0	0	0	0	0	0	0	0	0	0	0	0	0	0
?	002.50 - 002.54	456.247	2.51	1.80E-12	216424	185812	199629	0	0	0	0	0	0	0	0	0	327544	448623	379922	0	0	0	240395	230962	152759	348256	321949	262144	0	0	0
?	001.29 - 001.38	346.231	1.34	1.80E-12	158004	136203	185402	644548	546371	547914	0	0	0	0	231873	178597	545625	683268	549699	430890	572412	550553	649980	592786	652824	972688	853523	883215	0	0	0
?	001.76 - 001.76	516.318	1.78	2.00E-12	0	0	0	0	0	0	0	0	0	0	0	0	130689	186437	140606	0	0	0	0	0	0	0	0	0	0	0	0
?	001.45 - 001.53	301.153	1.47	2.30E-12	0	0	0	481087	459763	486620	0	0	0	0	0	0	212316	288985	195169	193408	228030	130752	213504	311187	376286	372281	392589	403645	0	0	0
?	001.63 - 001.68	555.626	1.66	2.40E-12	0	0	0	291725	263104	312320	0	0	0	0	0	0	163456	178112	219904	0	0	0	318150	262656	334720	456786	402452	584704	0	0	0
[20+H]+	000.99 - 001.25	330.236	1.18	2.60E-12	217647	210712	276342	1091874	1022397	1172683	133991	0	0	163670	143808	161191	409095	460745	504653	551570	638992	522121	861722	638903	871131	800806	821417	1044057	0	0	0
?	003.45 - 003.47	782.457	3.46	3.00E-12	0	0	0	256208	170702	215520	0	0	0	0	0	0	0	0	0	0	0	0	0	0	0	0	0	0	0	0	0
?	001.87 - 001.94	426.233	1.89	3.80E-12	0	0	0	368146	344287	428735	0	0	0	0	0	0	0	0	0	0	106420	0	153728	130560	178494	172254	186475	204845	0	0	0
Source MS fragment of 13?	002.06 - 002.09	380.125	2.08	4.40E-12	0	0	0	0	0	0	0	0	0	0	0	0	0	0	0	0	0	0	0	0	0	262144	312866	206464	0	0	0
?	002.99 - 003.04	514.262	3.02	4.50E-12	520768	524446	500014	303190	251480	207488	0	0	0	0	153088	0	463090	584949	506290	132140	0	0	452196	410413	401976	529414	516262	490196	0	0	0
?	002.67 - 002.71	566.261	2.70	4.60E-12	247185	285576	262336	183744	321850	301649	0	0	0	0	0	0	318812	417556	388816	0	0	0	283239	258176	291549	337420	404568	329678	0	0	0
?	003.92 - 003.98	687.410	3.98	4.60E-12	240019	162919	171165	0	0	0	0	0	0	170108	156229	207876	0	0	0	0	0	0	0	0	0	0	0	0	0	0	0
?	001.26 - 001.36	450.739	1.34	5.60E-12	144064	0	160512	130560	0	0	0	0	0	184337	179776	201125	812116	843330	912102	583035	891049	525881	268992	275550	272128	965049	1136203	1084176	0	0	0
Source MS fragment of 1?	002.30 - 002.33	268.166	2.33	5.70E-12	219968	315724	329875	0	0	0	0	0	0	0	0	0	0	0	0	0	0	0	0	0	0	0	0	0	0	0	0
?	001.39 - 001.55	413.272	1.50	5.80E-12	0	0	0	532544	568530	412754	0	0	0	0	0	0	239043	247504	249173	225177	191473	179582	544772	393682	368654	374418	393729	363089	0	0	0
?	003.30 - 003.33	482.372	3.31	6.10E-12	0	0	0	0	0	0	0	0	0	0	0	0	0	0	0	0	0	0	0	0	0	146688	220651	172982	0	0	0
?	001.25 - 001.39	316.222	1.33	6.20E-12	0	0	150592	630972	669336	560723	0	0	0	160256	156864	153166	356560	522570	387966	296865	292560	310257	532215	478981	612073	565827	571471	660866	0	0	0
?	002.79 - 002.82	379.243	2.81	6.30E-12	0	0	0	0	0	0	0	0	0	372138	389194	536136	0	0	0	0	0	0	0	0	0	0	0	0	0	0	0
?	001.38 - 001.55	350.204	1.42	6.50E-12	134447	0	0	512526	453889	424170	0	0	0	0	118187	0	370881	434150	369243	272256	309732	214980	417723	388493	428977	482045	539857	482433	0	0	0
[1+H+K]2+	002.31 - 002.35	431.704	2.34	7.30E-12	261862	366750	256793	0	0	0	0	0	0	0	0	0	0	0	0	0	0	0	0	0	0	0	0	0	0	0	0
[14+H]+	001.57 - 001.65	606.294	1.64	1.00E-11	373693	435493	317506	0	0	0	0	0	0	173216	0	0	402777	441626	465391	0	0	0	399314	386911	418395	173424	191677	218506	0	0	0
?	001.82 - 001.93	431.264	1.88	1.20E-11	0	0	0	260558	298793	347803	0	0	0	0	0	0	186880	253977	272830	176130	164652	259072	382254	292122	355892	251985	292151	294897	0	0	0
?	001.48 - 001.51	431.276	1.50	1.30E-11	0	0	0	188462	164983	120704	0	0	0	0	0	0	0	0	0	0	0	0	0	0	0	0	0	0	0	0	0
?	003.69 - 003.70	462.257	3.71	1.30E-11	0	0	0	120931	187392	186688	0	0	0	0	0	0	0	0	0	0	0	0	121216	138235	104128	0	0	0	0	0	0
?	001.41 - 001.48	458.745	1.47	1.50E-11	216192	261931	263918	0	0	0	0	0	0	0	0	0	579373	689184	814908	166848	150272	204102	133122	0	0	203456	271330	259629	0	0	0
?	001.39 - 001.44	612.368	1.42	1.80E-11	206277	211483	174448	0	0	138322	0	0	0	0	0	129132	608325	507570	576278	201970	160384	201627	0	0	0	316136	380593	386660	0	0	0
?	001.77 - 001.79	597.302	1.78	1.80E-11	156800	238008	177024	0	0	0	0	0	0	0	0	0	0	0	0	0	0	0	0	0	0	0	0	0	0	0	0
[5-Me+2H]2+	001.96 - 001.99	391.205	1.97	1.90E-11	470365	729508	568028	0	0	0	0	0	0	0	0	0	0	0	0	0	0	0	0	0	0	0	0	0	0	0	0
[12+2H]2+	001.87 - 001.91	421.719	1.89	2.10E-11	289024	282880	265344	0	0	0	0	0	0	0	0	0	0	0	0	0	0	0	0	0	0	472512	688108	459648	0	0	0

[a] The dataset was generated from triplicate LC-MS data using Profiling Solution 1.1 (Shimadzu). All species that appeared in either *ΔbtmD* or unmodified production medium were removed, and species are ordered by significance (P-value). A full dataset is available as a spreadsheet from the corresponding author.

2. Supplementary Figures

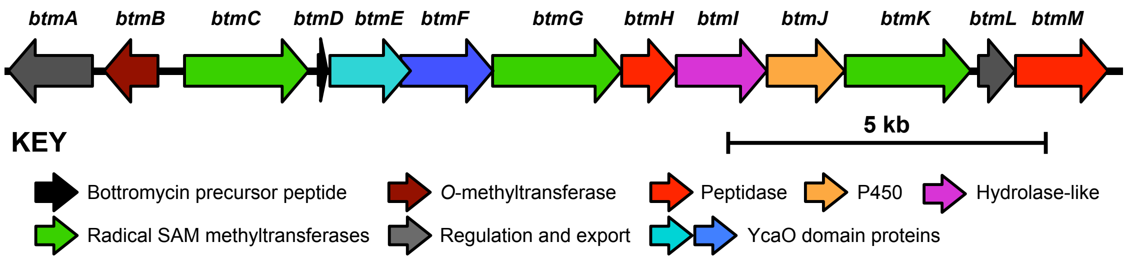

Figure S1 Bottromycin gene cluster in *S. scabies*.

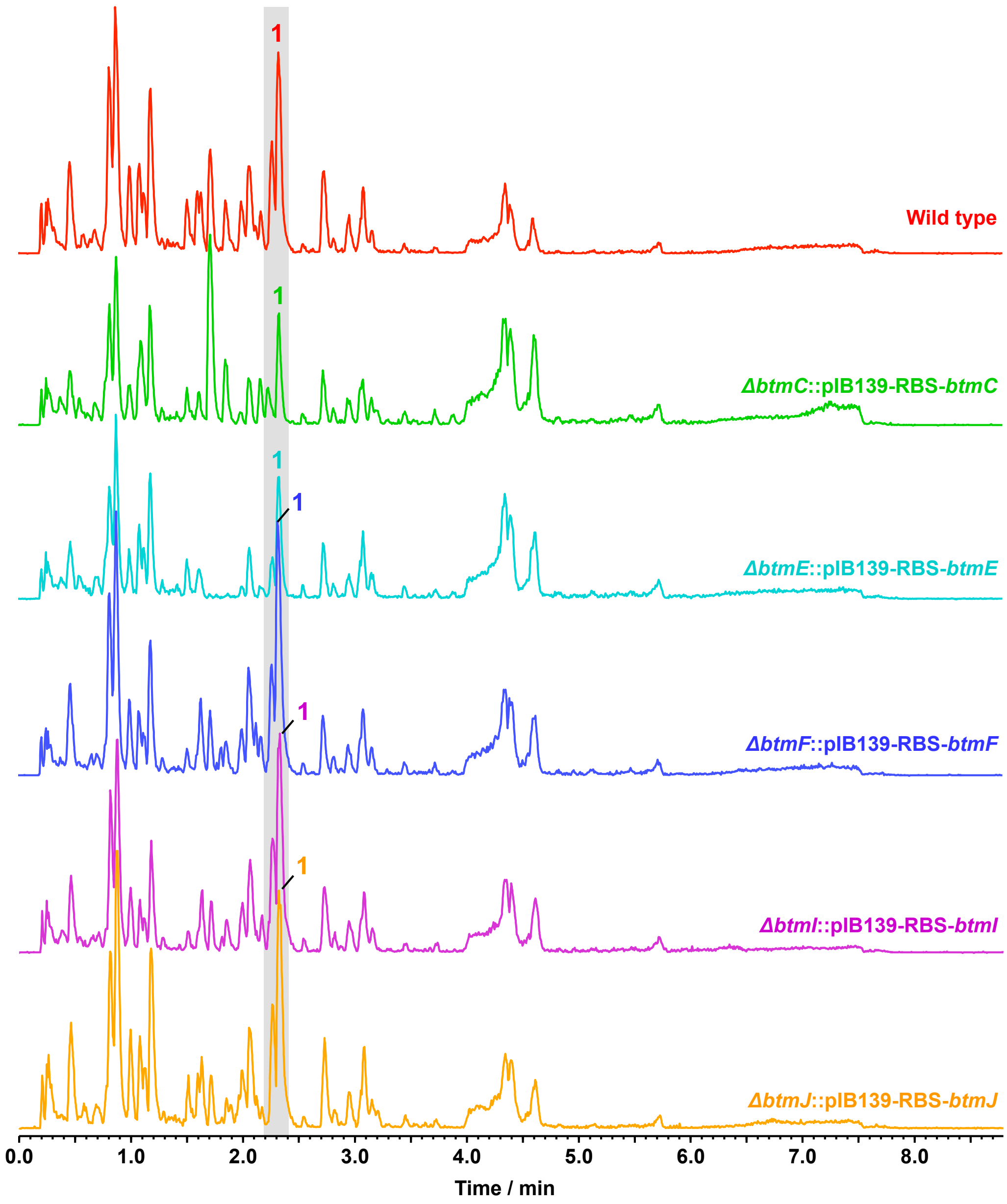

Figure S2 MS base peak chromatograms (BPCs) for all complemented strains compared to wild type *S. scabies* after 7 days in production medium. Bottromycin A_2 (**1**) production is highlighted.

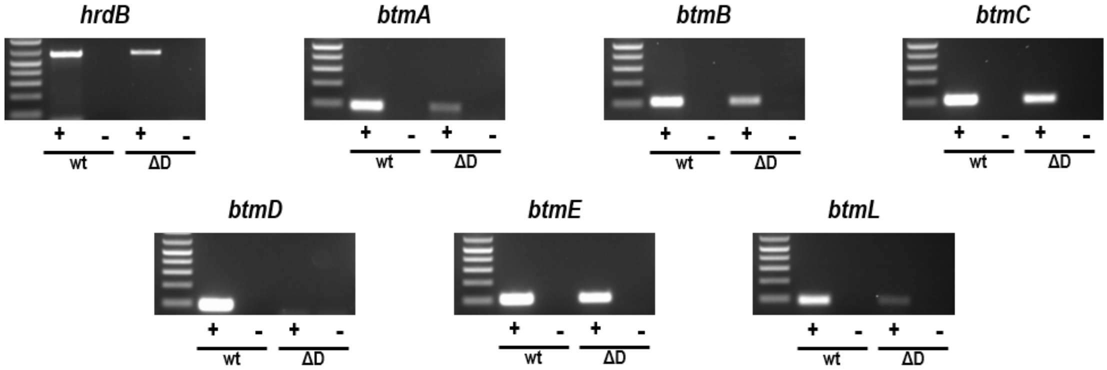

Figure S3 RT-PCR analysis of the wild type (WT) and *ΔbtmD* (ΔD) strains. Expression of several genes of the *btm* cluster (*A*, *B*, *C*, *D*, *E* and *L*) was assessed in 72h cultures of these strains, including negative controls (-) to check possible DNA contamination in the samples, and using *hrdB* as a positive control for gene expression.

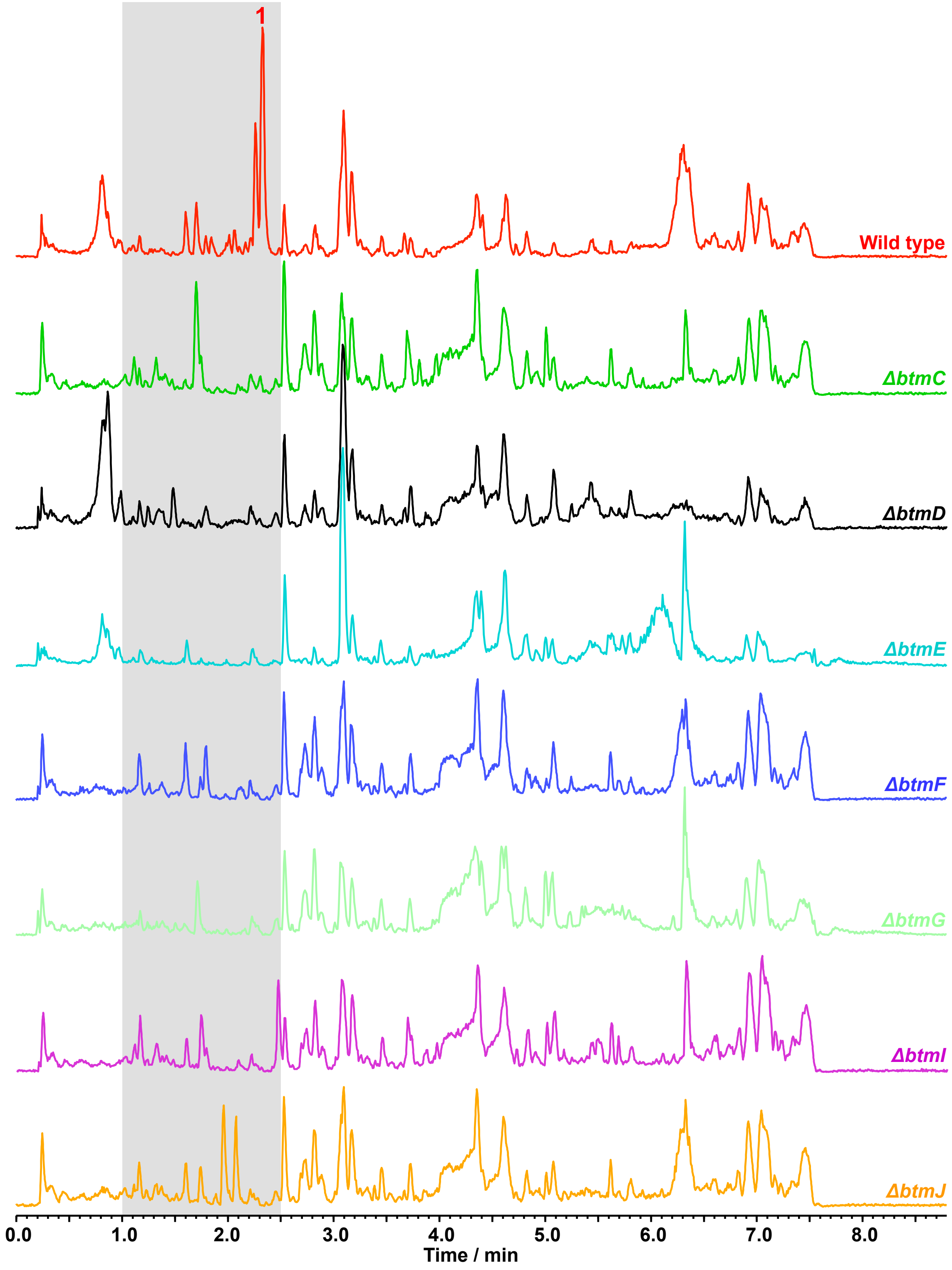

Figure S4 MS base peak chromatograms (BPCs) for all mutant strains compared to wild type *S. scabies* after 3 days in production medium. Bottromycin A_2 (**1**) production is indicated for the WT and the grey box indicates the region in which all bottromycin-like compounds eluted.

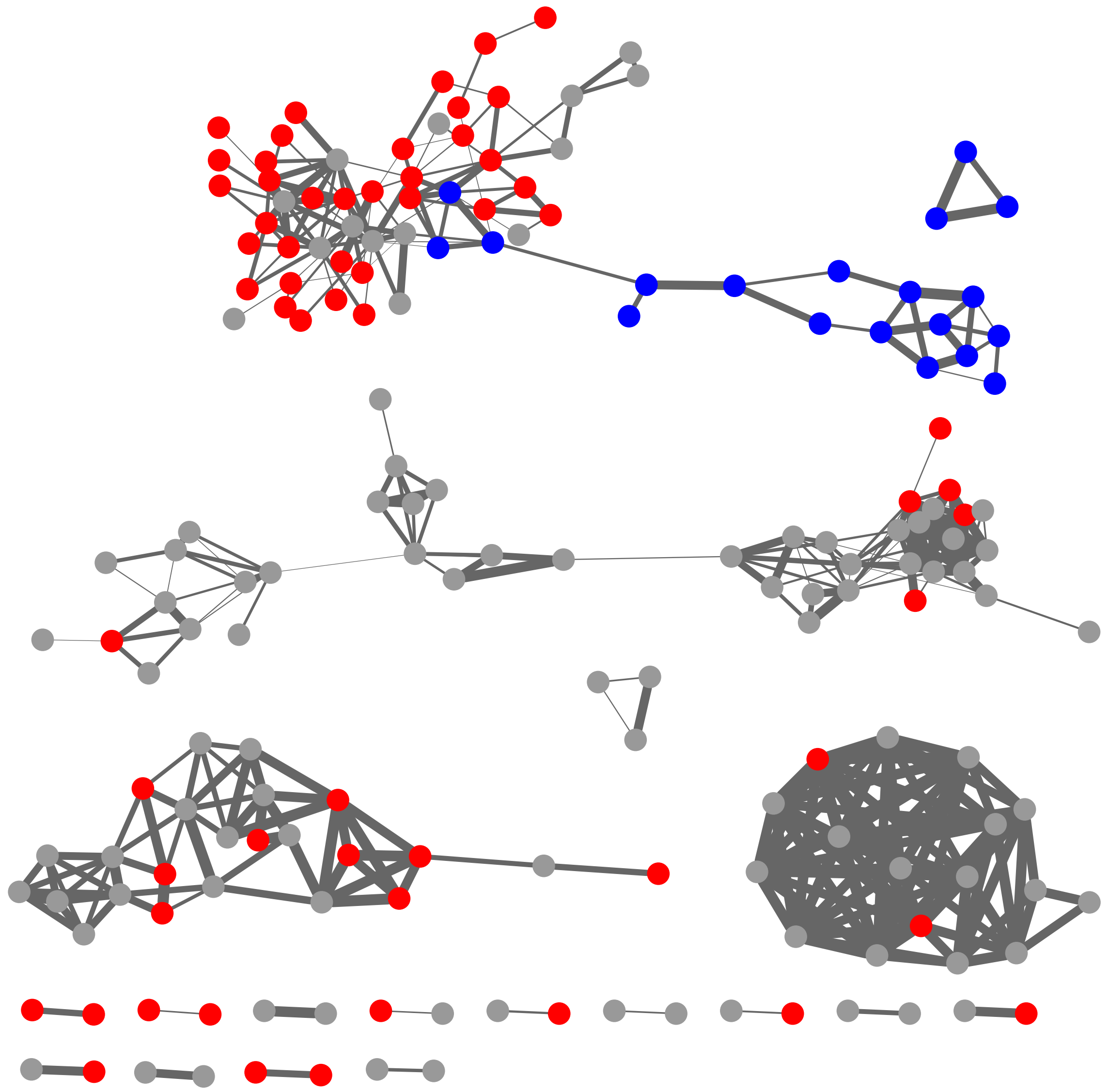

Figure S5 Mass spectral network view of the metabolomes of wild type (WT) *S. scabies* and *ΔbtmC*, *ΔbtmD*, *ΔbtmF*, *ΔbtmI* and *ΔbtmJ* mutant strains, generated using Cytoscape 2.8.3. All red nodes are species observed in one or more of the WT, *ΔbtmC*, *ΔbtmF*, *ΔbtmI* and *ΔbtmJ* strains, but not in *ΔbtmD*. The blue nodes refer to the bottromycin-related species identified in this study. Edge thickness reflects how closely related each node is (a linear gradient of 2 to 40 points between cosine 0.6 and 1). Nodes not found in *ΔbtmD* that are in other areas of the network could either reflect a degree of natural variation between MS data or as-yet-uncharacterized metabolites related to the bottromycin pathway.

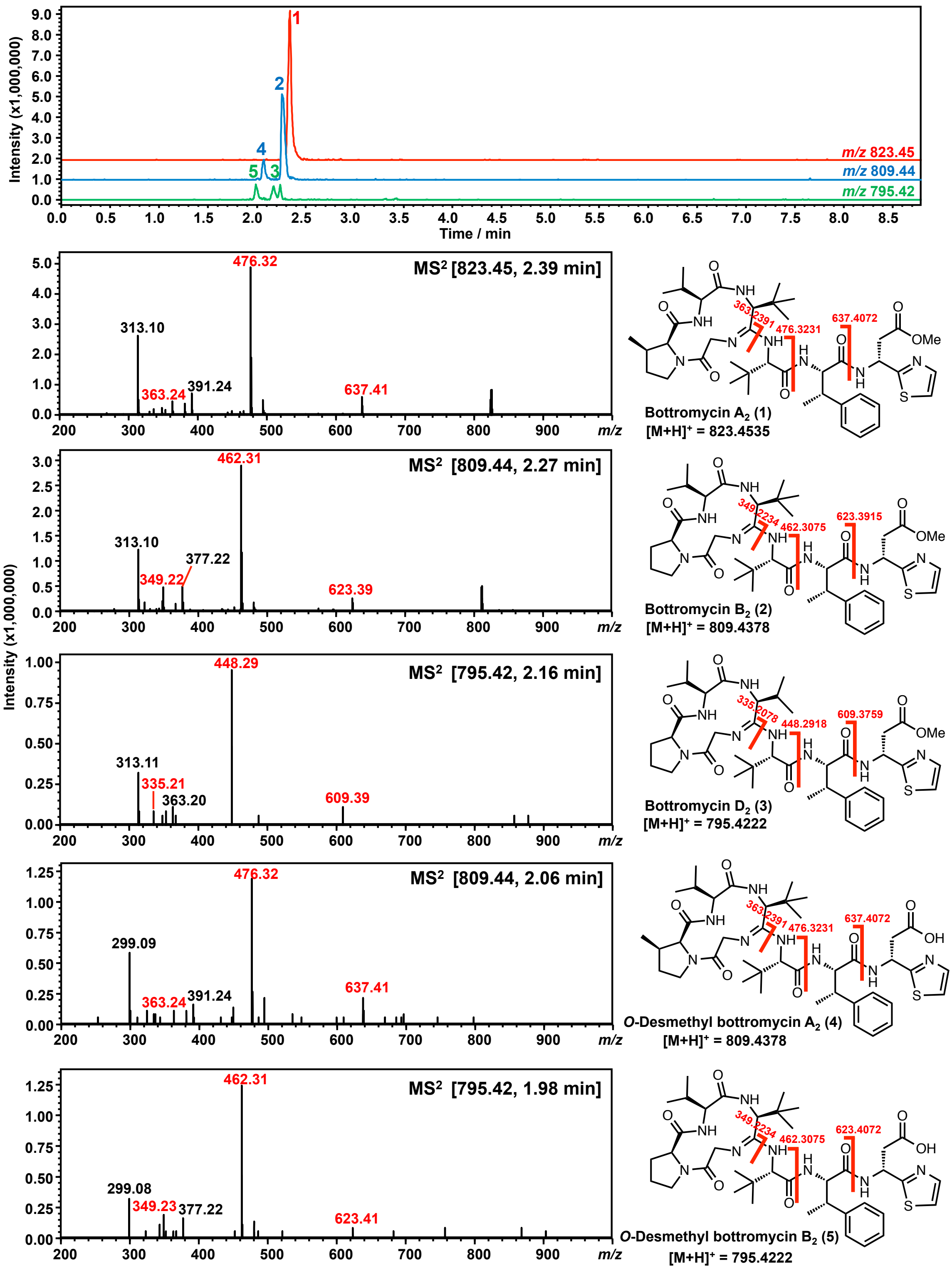

Figure S6 LC-MS^2 data for mature bottromycins **1**, **2** and **3**, and *O*-desmethyl bottromycins (DMBs) **4** and **5**. All data is from wild type *S. scabies*.

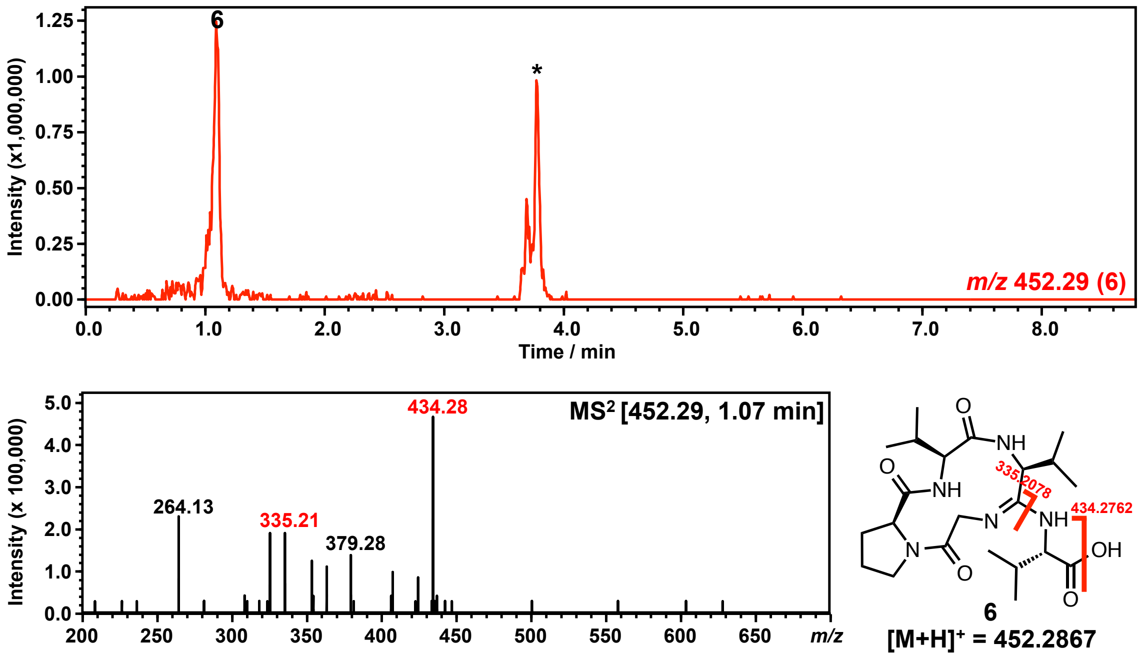

Figure S7 LC-MS^2 data for truncated cyclized metabolite **6**. The starred peak is an unrelated metabolite with the same mass as **6**. Data is from *S. scabies ΔbtmC*.

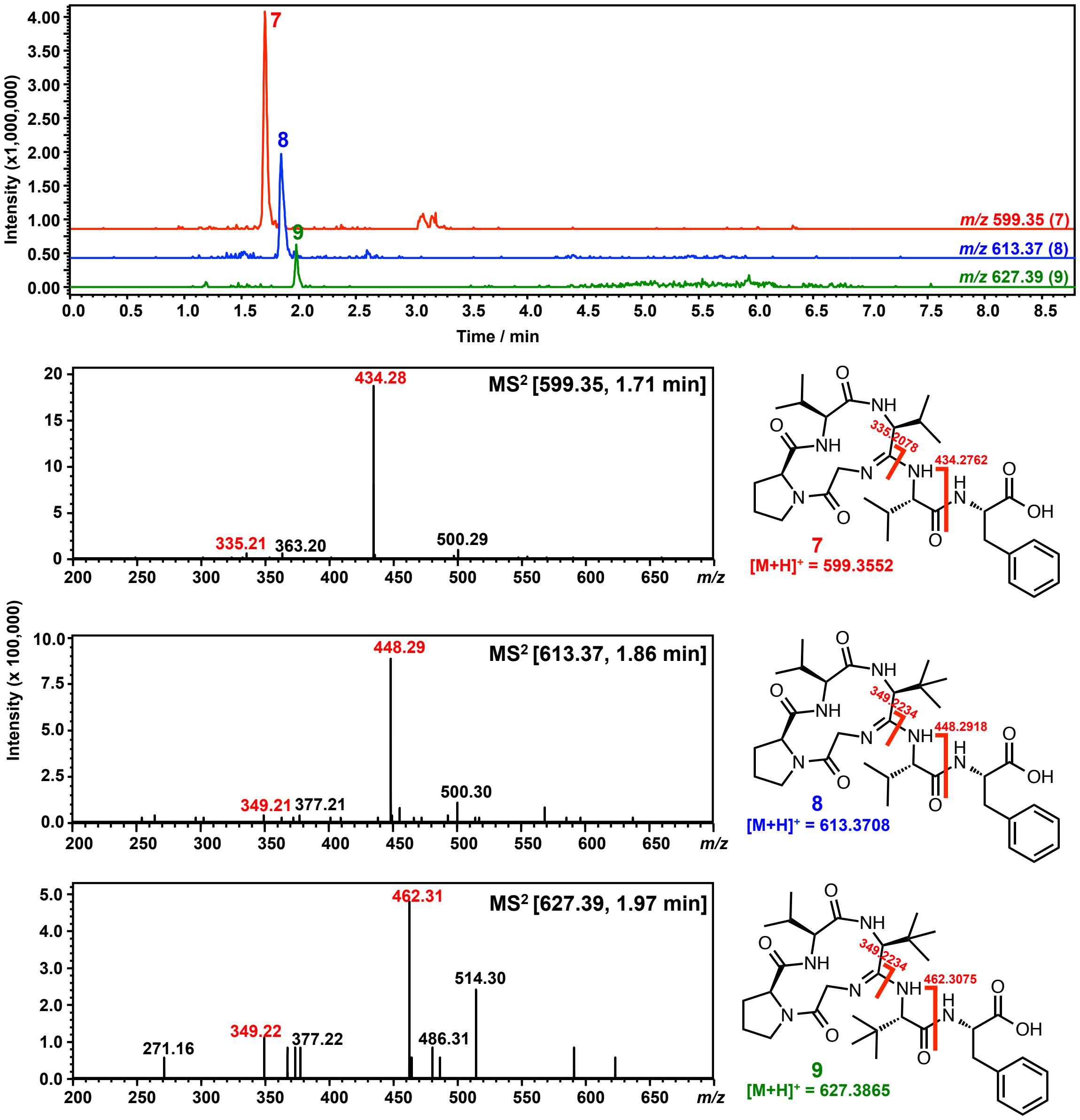

Figure S8 LC-MS^2 data for truncated cyclized metabolites **7**, **8** and **9**. All data is from wild type *S. scabies*.

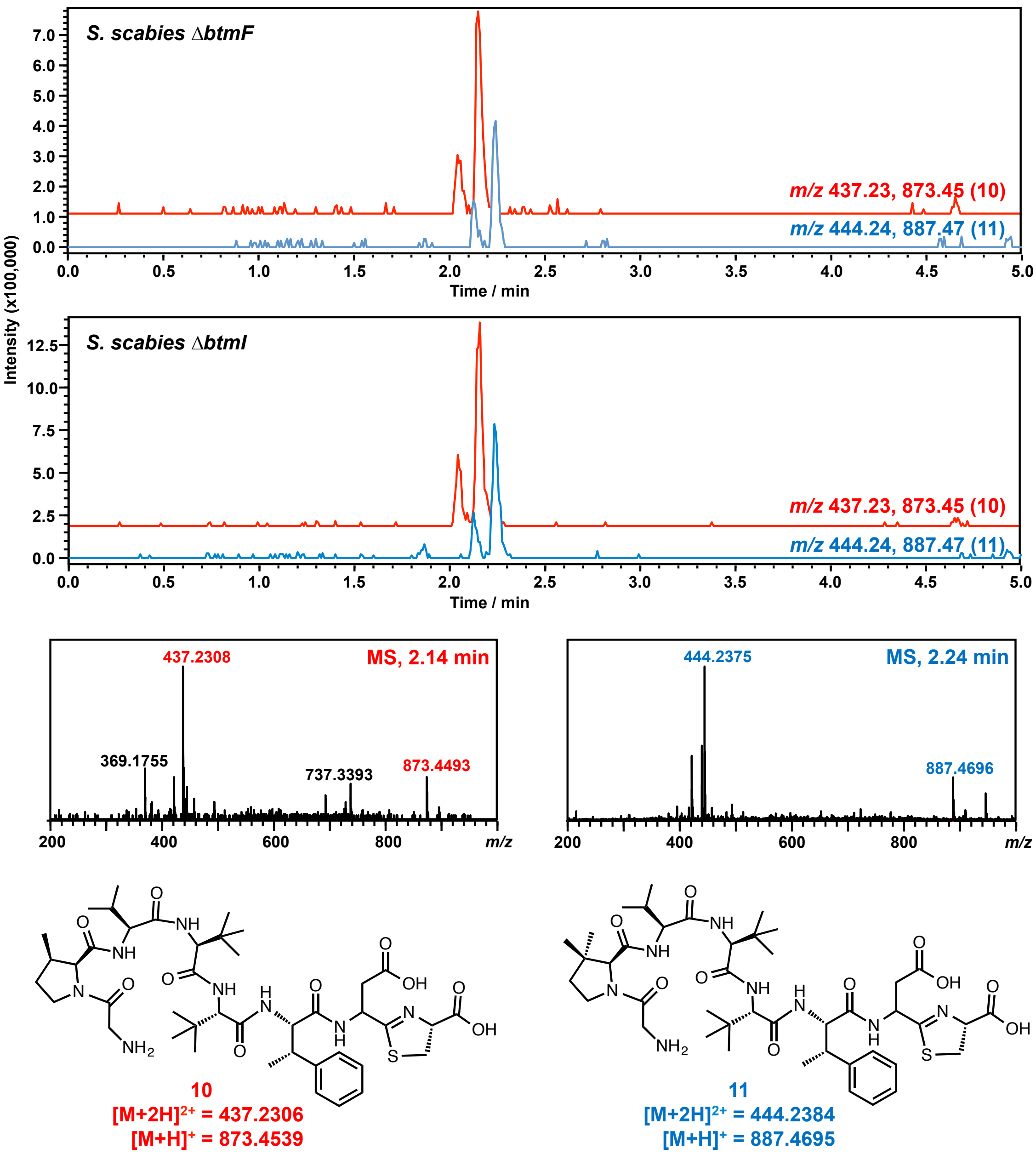

Figure S9 LC-MS data for linear metabolites **10** and **11** produced by *S. scabies ΔbtmF* and *ΔbtmI*.

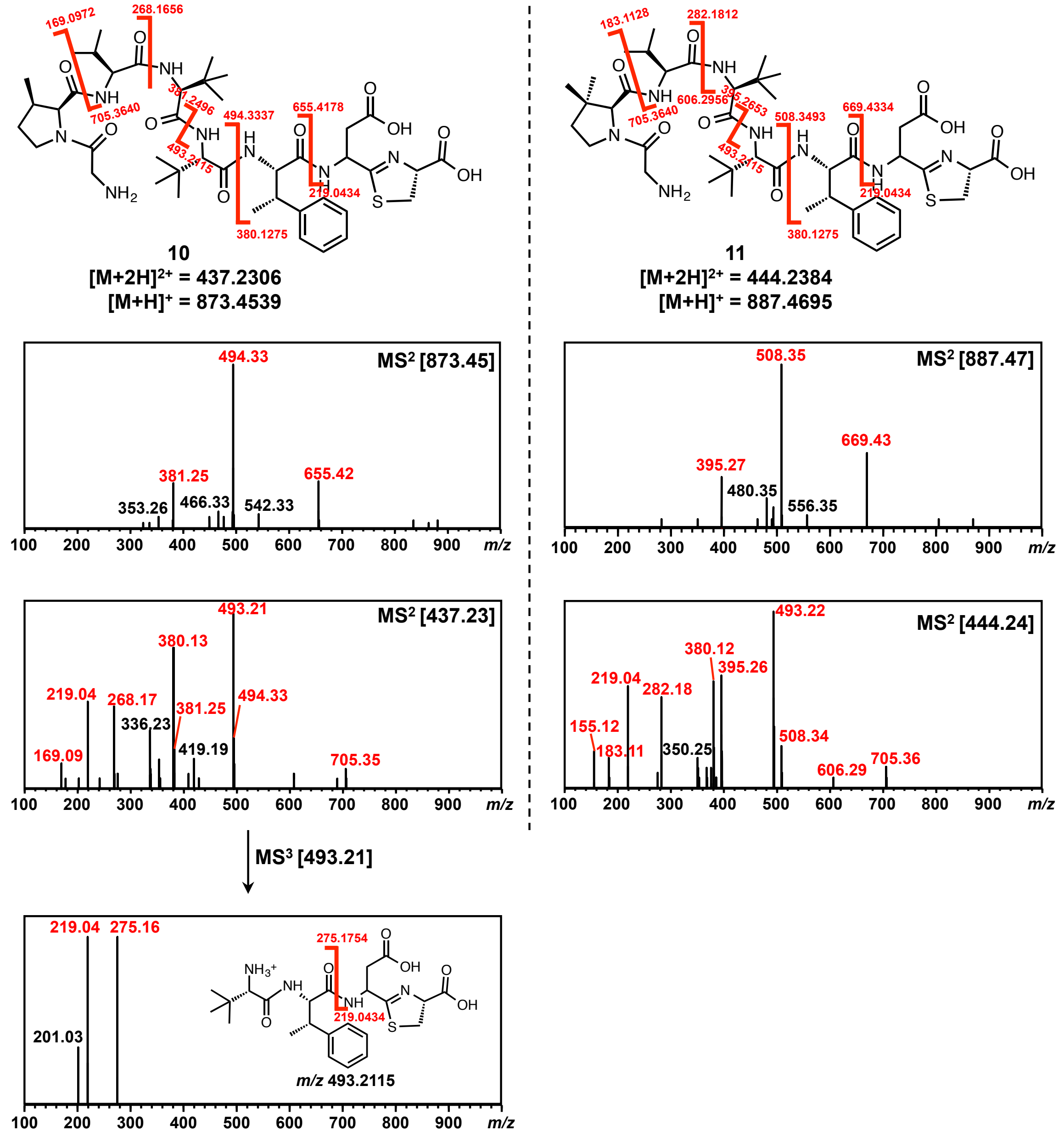

Figure S10 MS^2 and MS^3 data for **10** and **11** from *S. scabies ΔbtmI*.

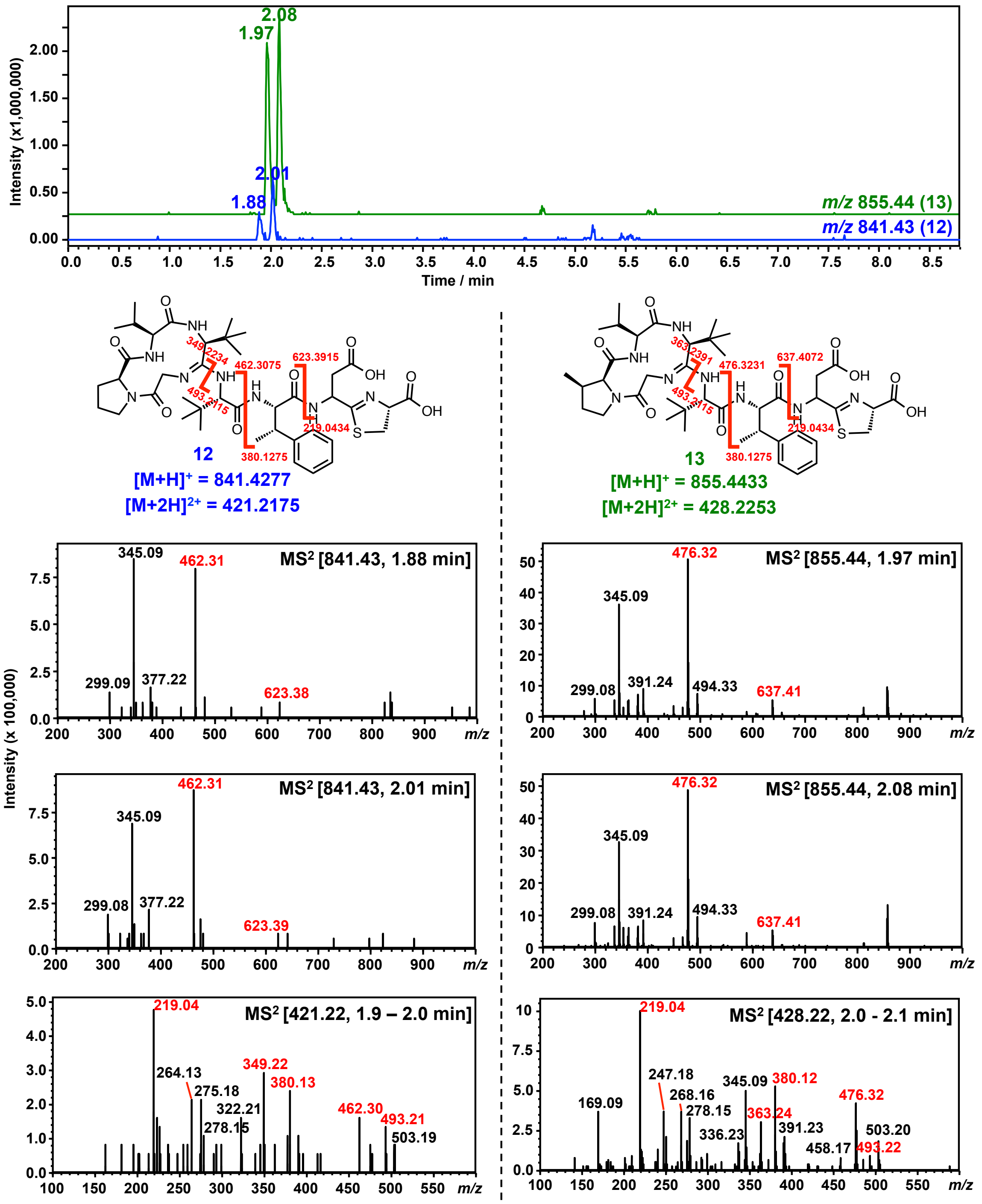

Figure S11 LC-MS^2 data for carboxylated bottromycins (CBs) **12** and **13**. All data is from *S. scabies ΔbtmJ*.

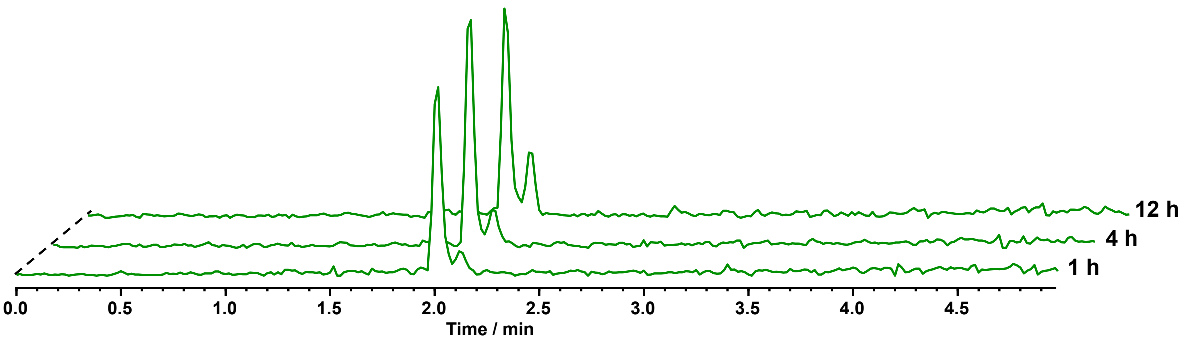

Figure S12 Time course showing the conversion of a single purified peak of **13** into two peaks with identical masses. Extracted ion chromatograms from LC-MS analysis are shown.

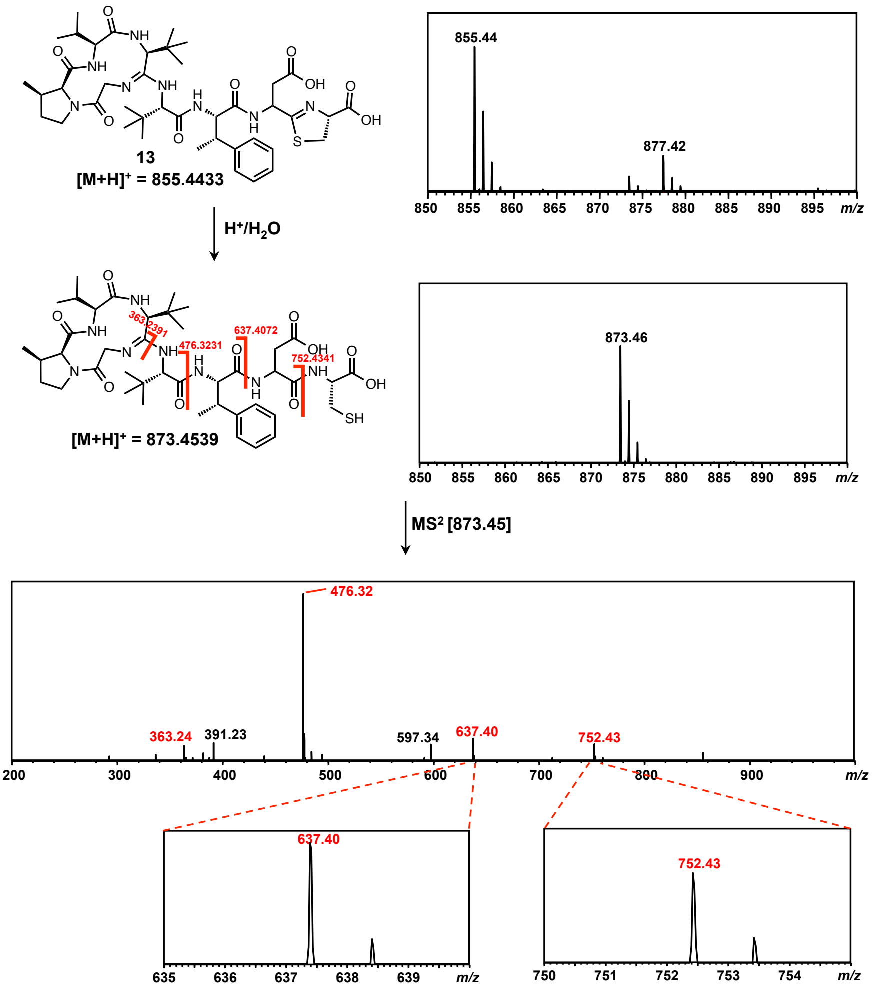

Figure S13 LC-MS^2 data for carboxylated bottromycin **13** treated with 0.1% aqueous formic acid, which hydrolyzes the thiazoline back to a cysteine residue. This conversion is characterized by an increase of 18.01 Da to the mass of the compound and the *m/z* 752.43 fragment in the MS^2 spectrum. This distinguishes this compound from **10**, which has an identical mass but retains the thiazoline and instead has no amidine.

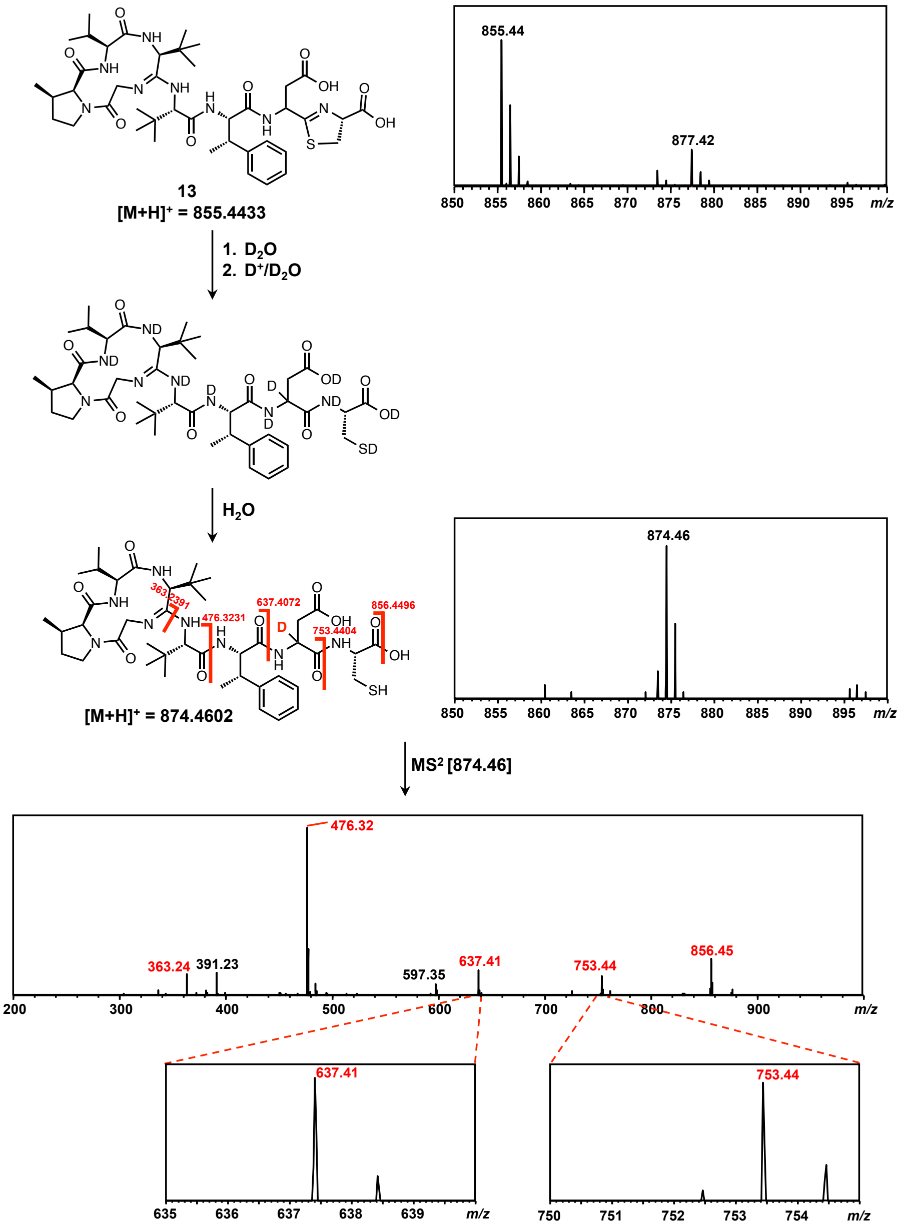

Figure S14 Deuterium exchange showing aspartate epimerization in carboxylated bottromycin **13**. LC-MS^2 data is shown for **13** treated sequentially with: (1) D_2O and (2) 40 mM DCl in D_2O; and then H_2O. The D_2O steps first lead to the replacement of protons with deuteriums in all readily exchangeable positions and acid treatment hydrolyses the thiazoline residue as shown in Fig. S13. Following hydrolysis, back exchange of all exchangeable deuteriums takes place in H_2O. However, the deuterium in the α-position of aspartate is trapped, as there is no longer an adjacent thiazoline to enable exchange. The specific incorporation of one deuterium is shown by the increase in one mass unit in the full molecule, while the location of this deuterium is demonstrated by the *m/z* 753.44 and *m/z* 637.41 fragments.

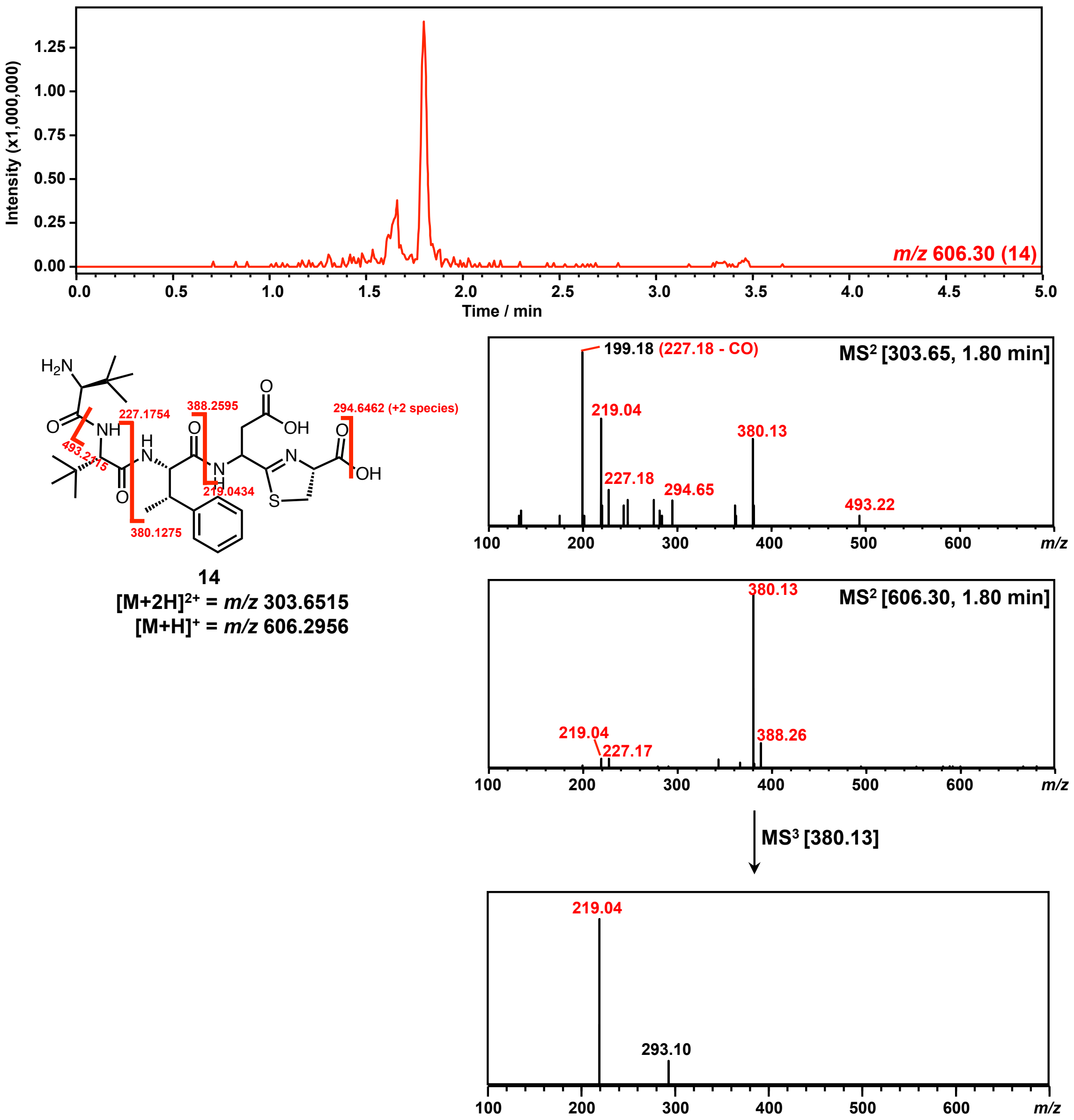

Figure S15 LC-MS^n data for **14** produced by wild type *S. scabies*.

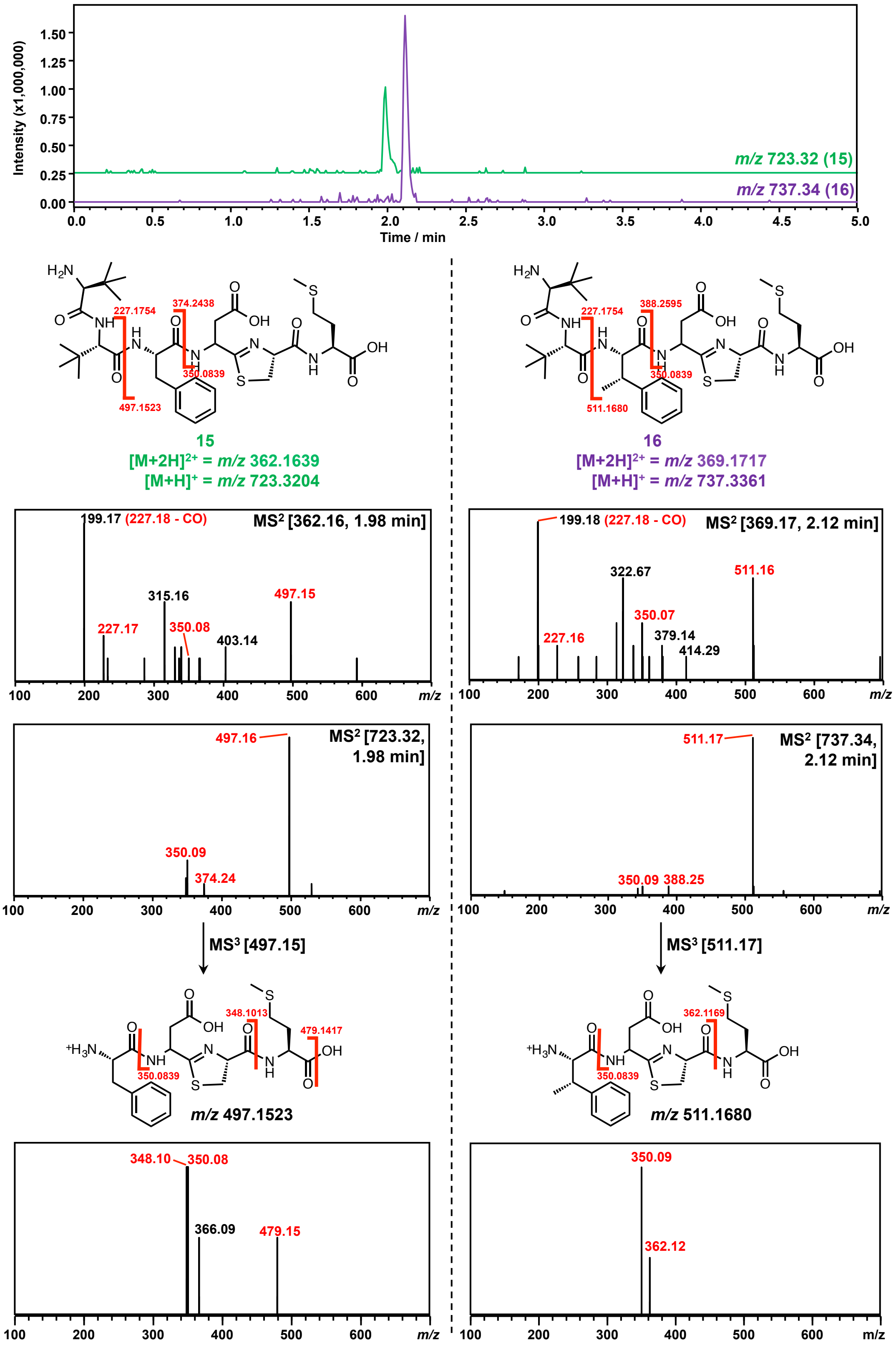

Figure S16 LC-MS^n data for **15** and **16** produced by wild type *S. scabies*.

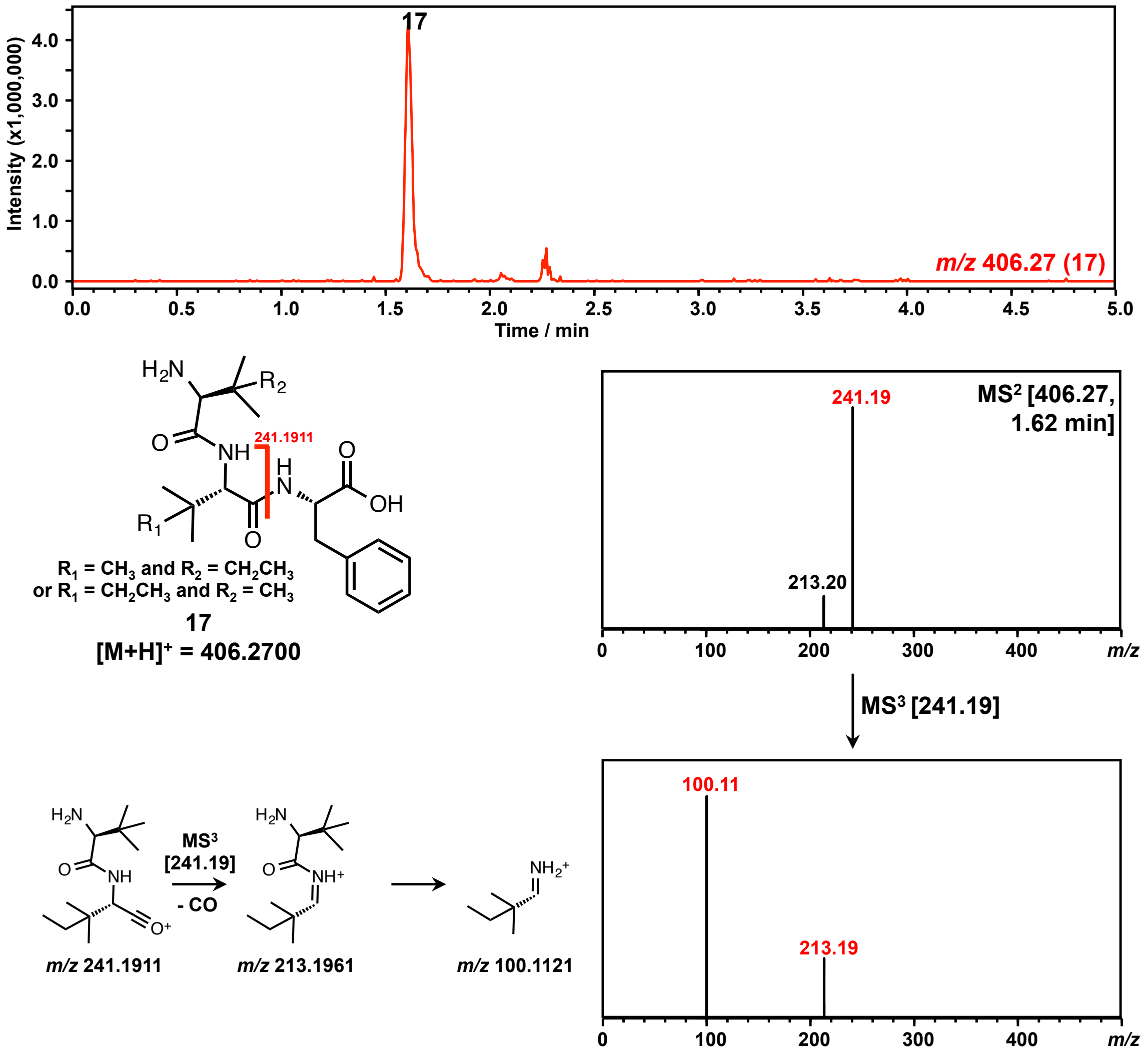

Figure S17 LC-MS^n data for **17** produced by *S. scabies ΔbtmC*. A fragmentation pathway for *m/z* 241.19 is proposed, although an alternative fragmentation route could place the additional methyl group on the other valine.

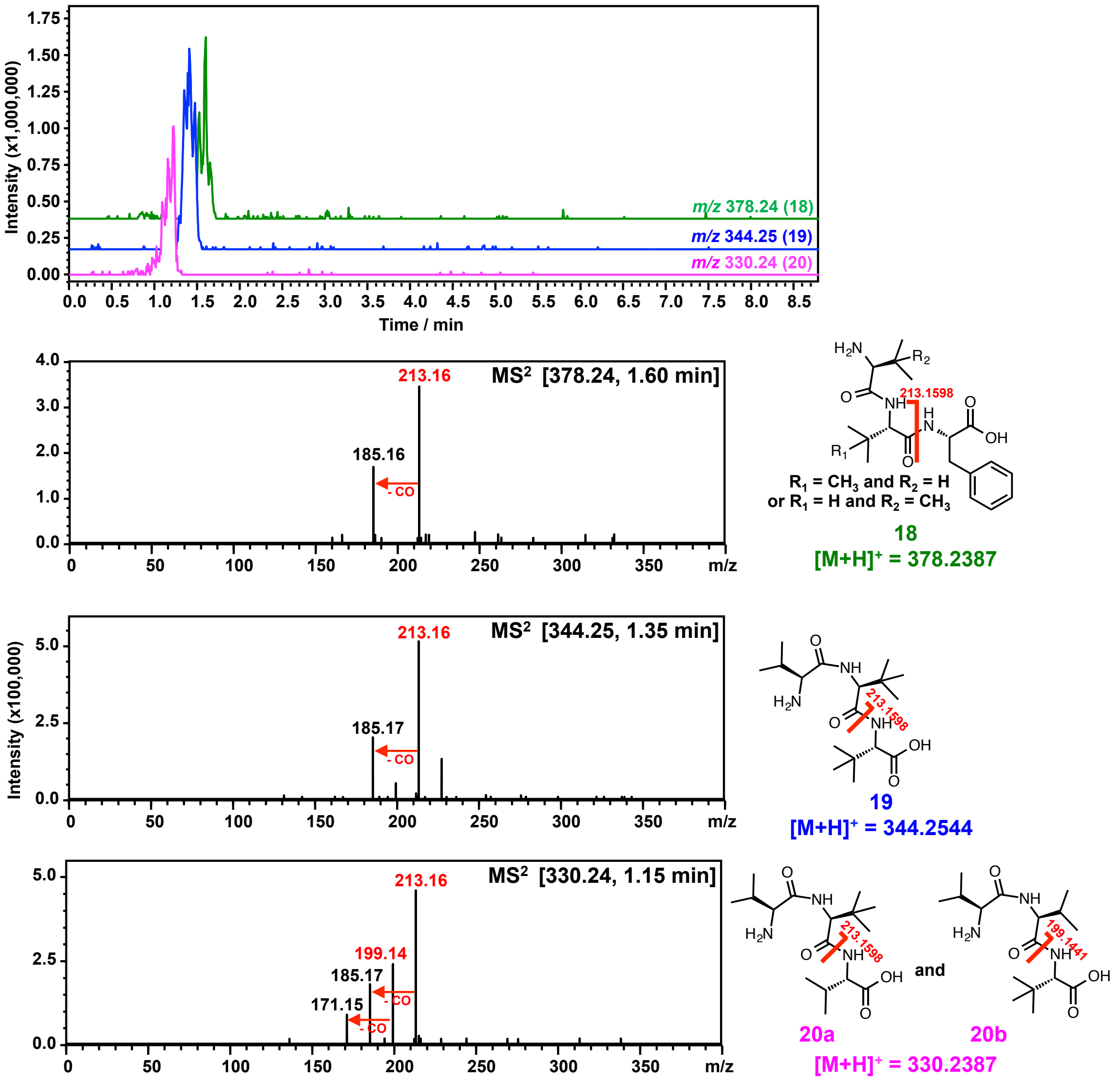

Figure S18 LC-MS^2 data for peptide metabolites deriving from the middle of the bottromycin core peptide. All data is from *S. scabies ΔbtmC*. Loss of CO from a peptide b ion results in a loss of 27.99 Da.

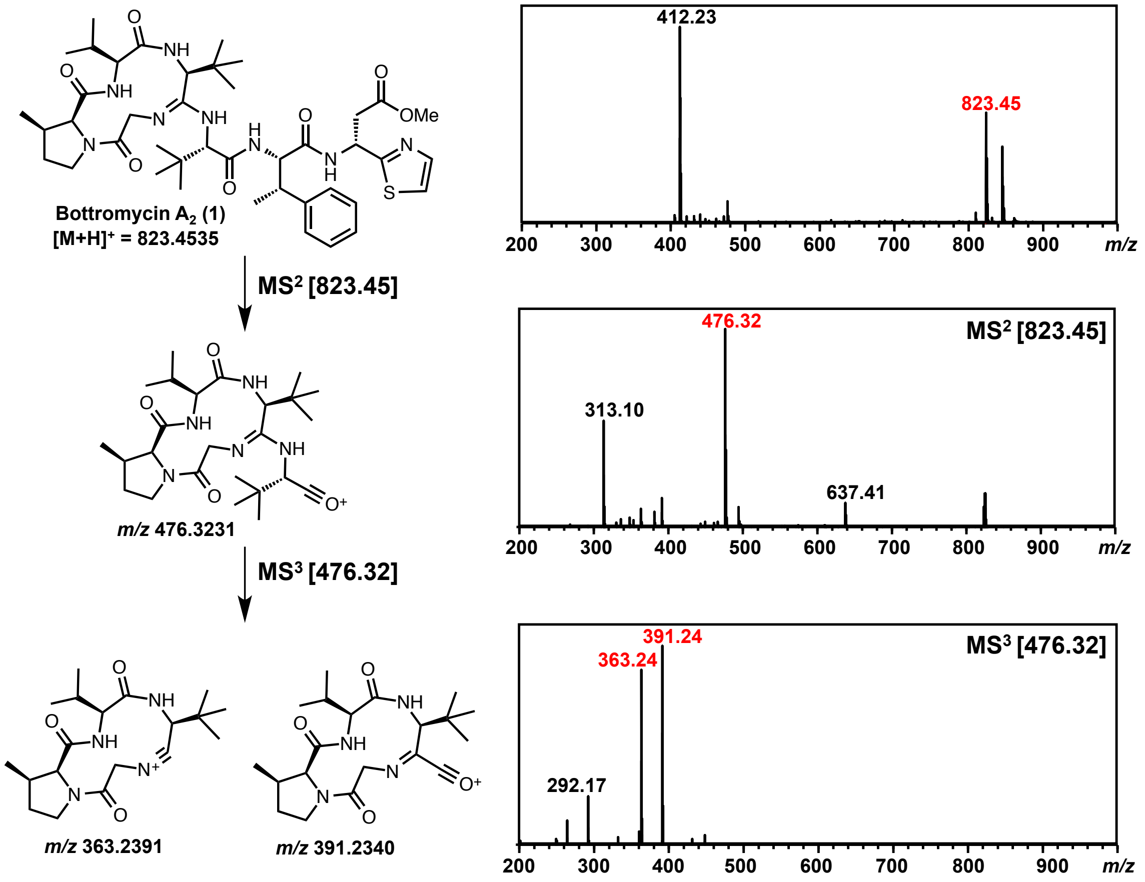

Figure S19 LC-MS^n data for bottromycin A_2 (**1**) showing the production of a fragment of *m/z* 476.32 that is consistent with a CO adduct of the nitrilium ion (*m/z* 391.2340). This could form by fragmentation to give CO, an imine and the nitrilium ion followed by recombination of the CO and nitrilium ion before they separate. This MS^n pattern is characteristic for metabolites that feature the macrocyclic amidine.

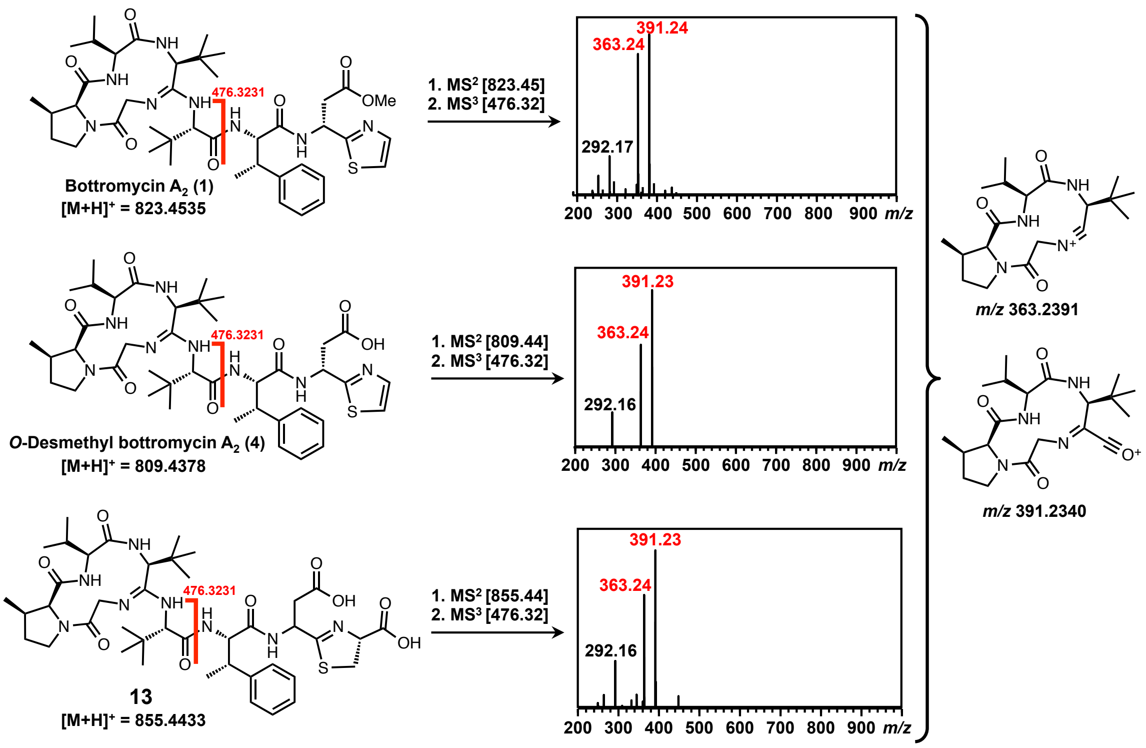

Figure S20 LC-MS^n data for molecules featuring the same macrocyclic amidine as bottromycin A_2 (**1**). These all provide an MS^3 pattern that is consistent with the fragmentation pathway detailed in Fig. S19.

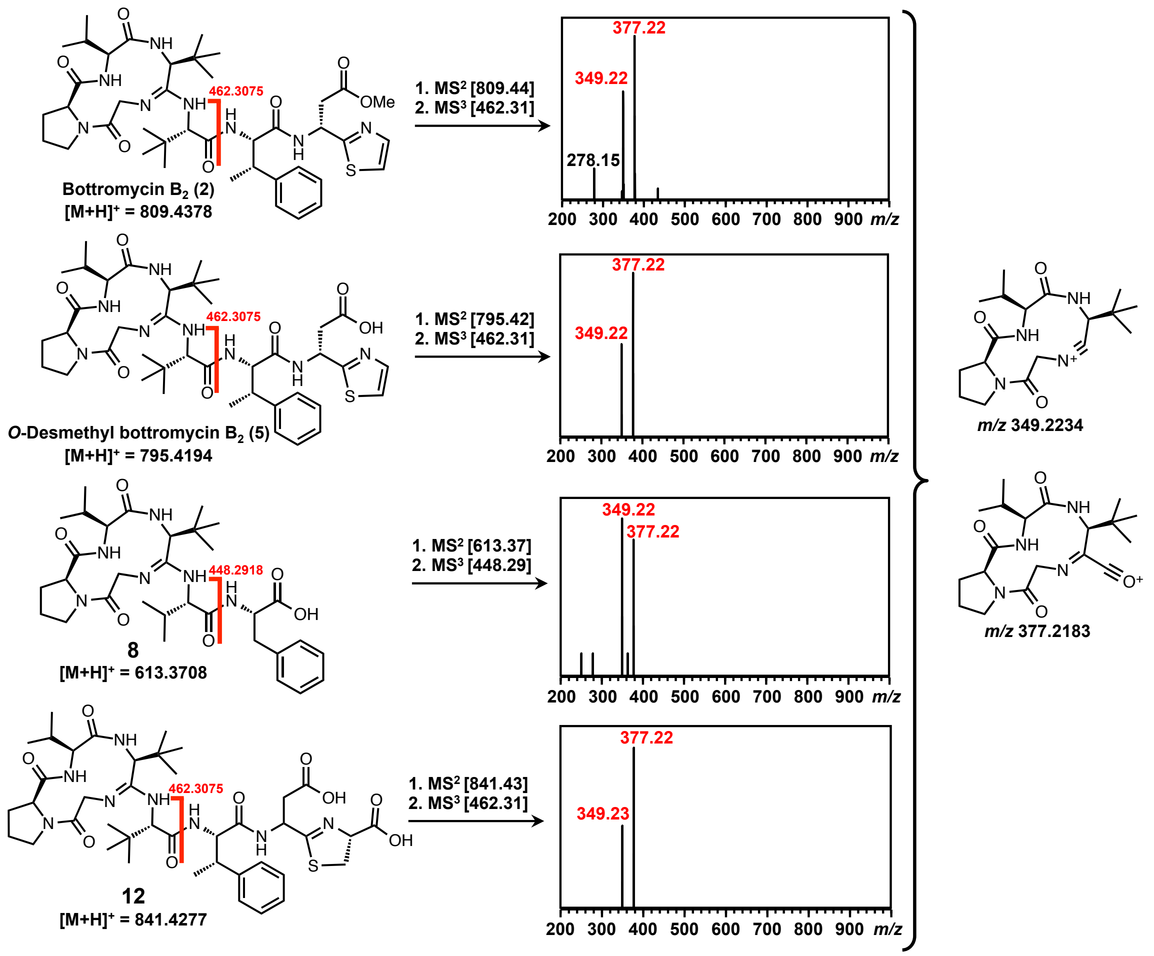

Figure S21 LC-MS^n data for molecules featuring the same macrocyclic amidine as bottromycin B_2 (**2**). These all provide an MS^3 pattern that is consistent with the fragmentation pathway detailed in Fig. S19.

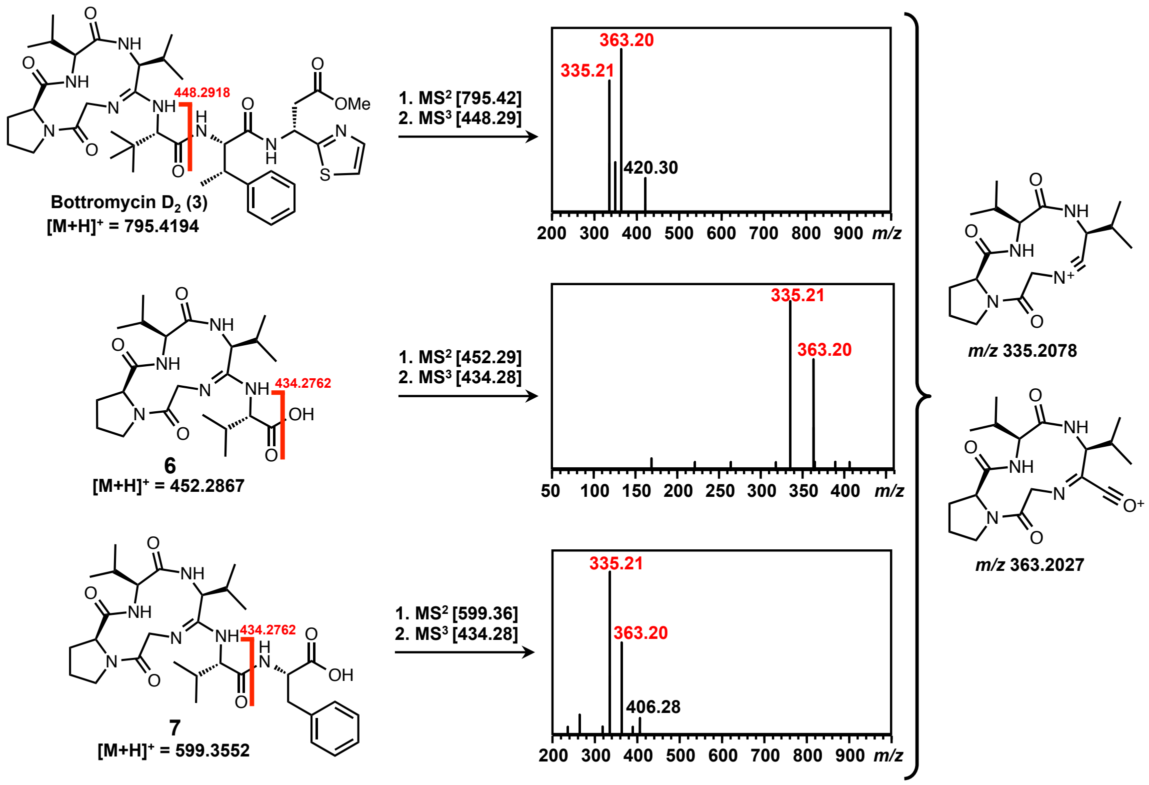

Figure S22 LC-MS^n data for molecules featuring the same macrocyclic amidine as bottromycin D_2 (**3**). These all provide an MS^3 pattern that is consistent with the fragmentation pathway detailed in Fig. S19.

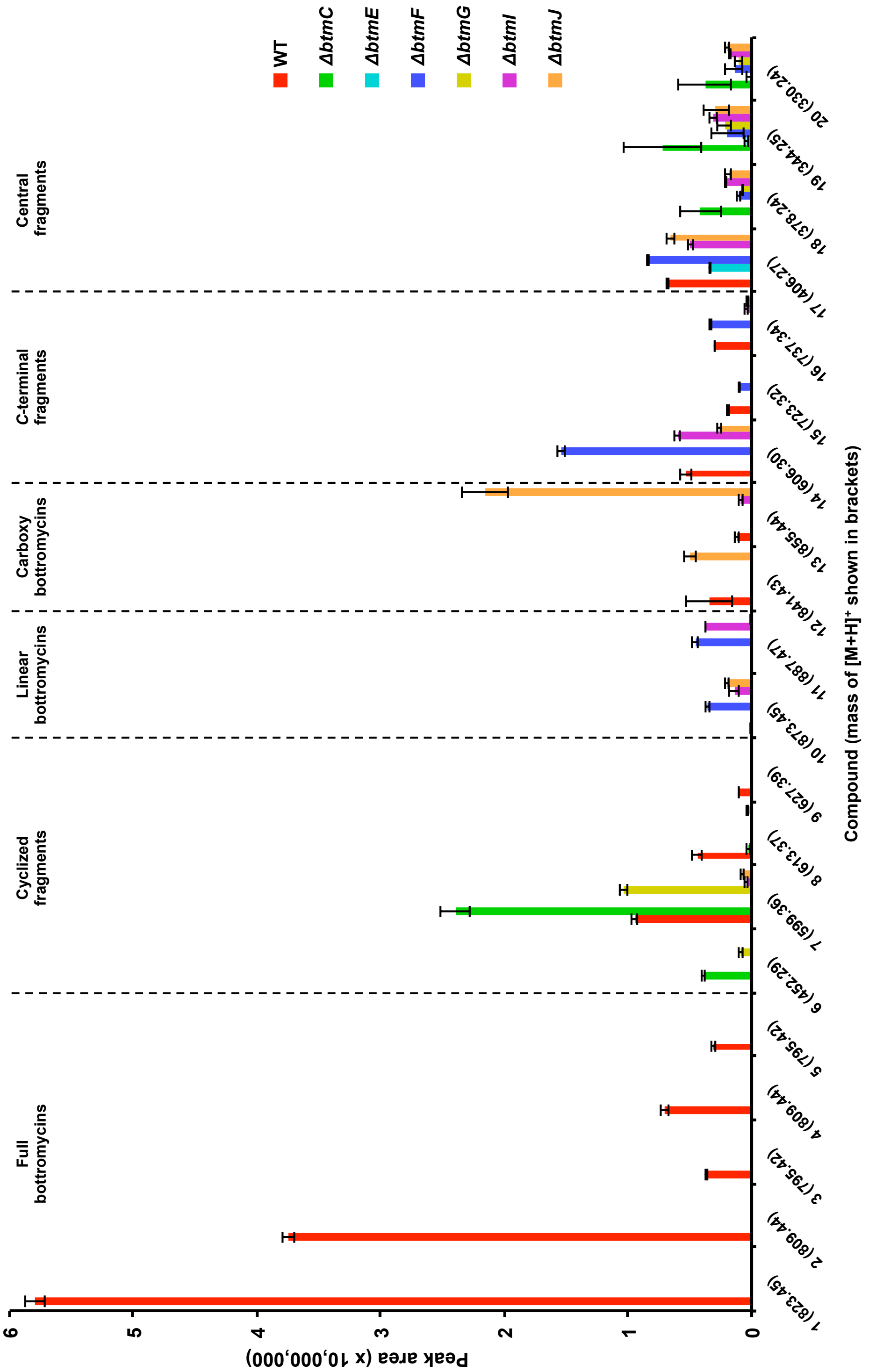

Figure S23 Chart showing the relative proportion of metabolites produced across all mutants after three days in production medium. The quantities represent the average of triplicate datasets and reflect the MS peak areas of the summed $[M+H]^+$ and $[M+2H]^{2+}$ signals for each compound. Error bars represent the standard deviation of triplicate data.

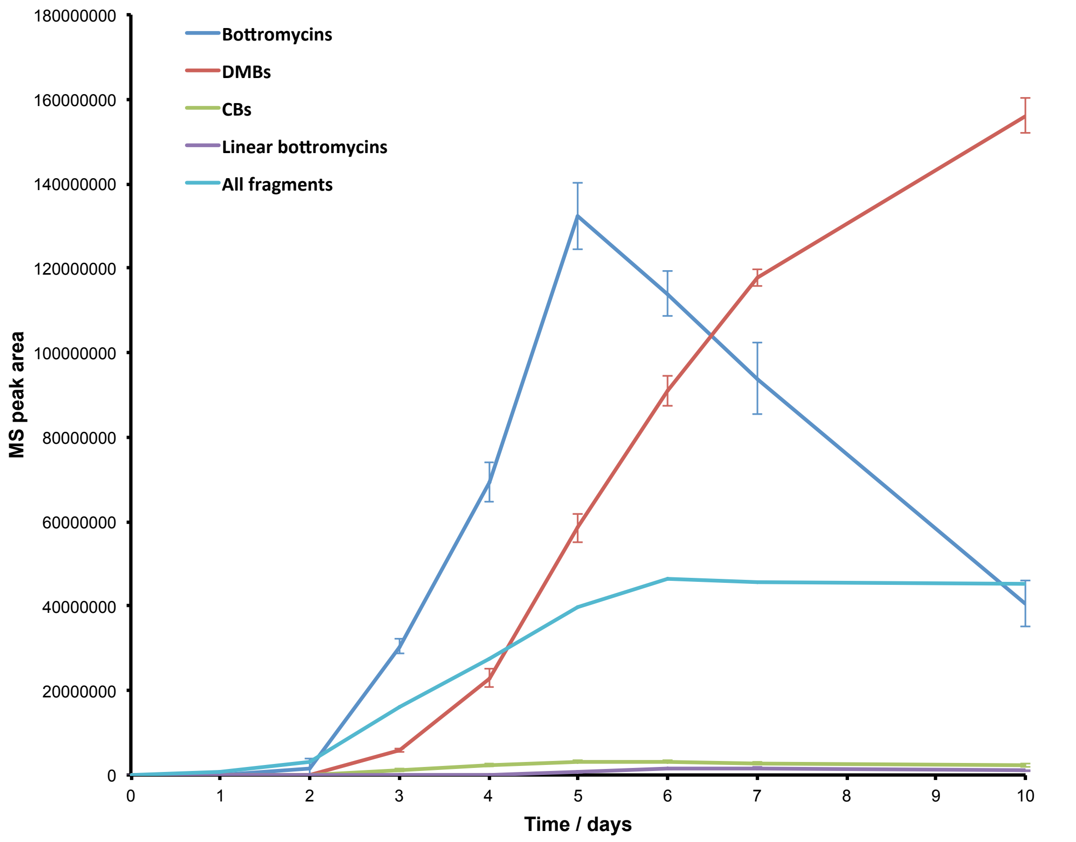

Figure S24 Time-course analysis of bottromycins produced by wild type *S. scabies*. Error bars represent the standard deviation of triplicate data. Molecules have been grouped as: (i) bottromycins (**1**, **2** and **3**), (ii) DMBs (*O*-desmethyl bottromycins, **4** and **5**), (iii) CBs (carboxy bottromycins, **12** and **13**), (iv) Linear bottromycins (**10** and **11**), (v) truncated bottromycin fragments (**7**, **8**, **9**, **14**, **15** and **16**). Small amounts of linear bottromycins only appear after 6 days, so do not feature as wild-type metabolites in Figures 1 and S23, which are based on data collected after 3 days.

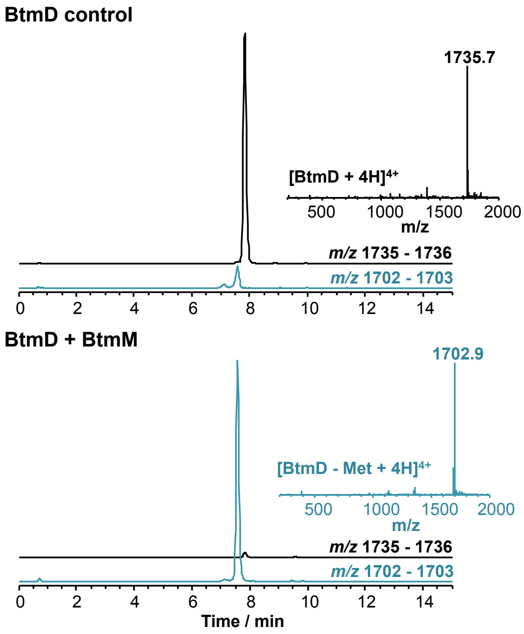

Figure S25 BtmM activity with BtmD. *N*-terminal methionine cleavage from *C*-terminally His_6-tagged BtmD is catalyzed by BtmM. MS data from 7 - 8 min is shown. The molecular weight of BtmD-His_6 is 6,937.6 Da (calc. $[M+4H]^{4+}$ m/z = 1,735.4; calc. $[M\text{-}Met+4H]^{4+}$ m/z = 1,702.6).

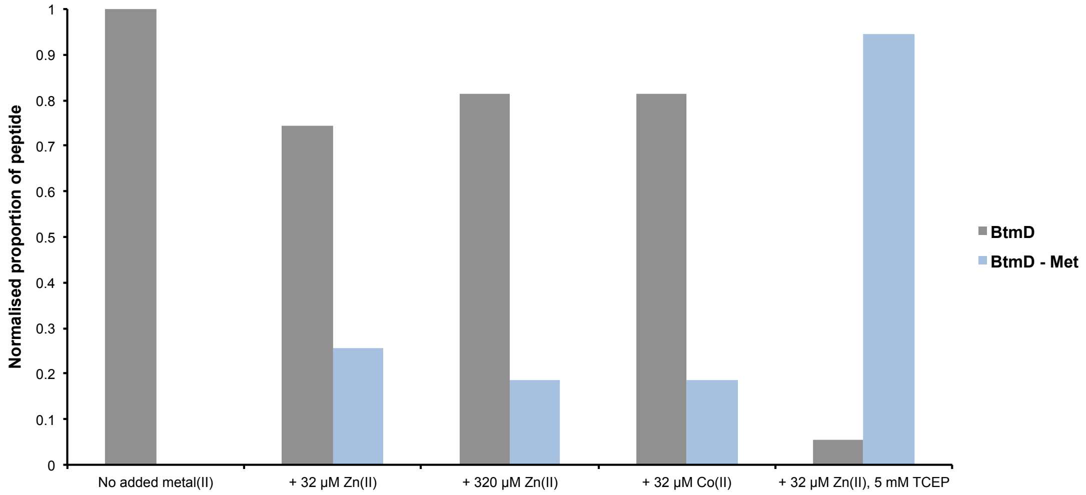

Figure S26 The efficiency of BtmD methionine cleavage by BtmM with various additives. Standard reaction conditions were 6.4 µM BtmM, 50 mM Tricine pH 9, 30 °C for 3 hours.

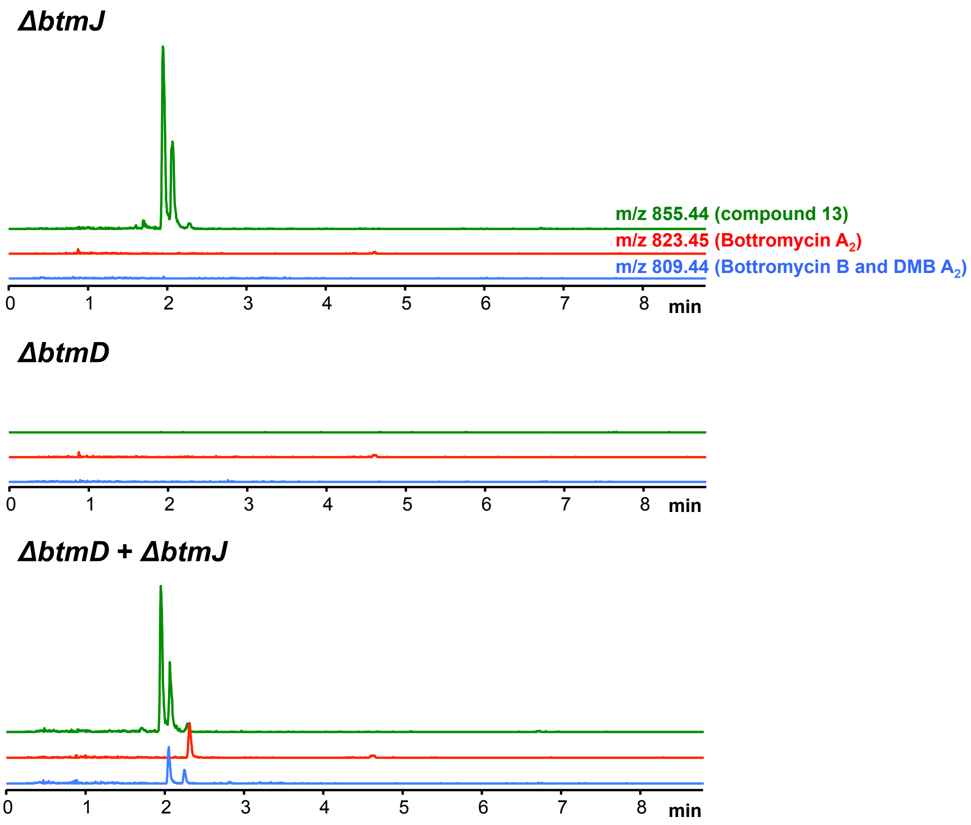

Figure S27 LC-MS analysis of *S. scabies ΔbtmJ* + *ΔbtmD* co-culture. Traces show extracted ion chromatograms for *m/z* 855.44, 823.45 and 809.44 in *ΔbtmJ* only, *ΔbtmD* only and in the co-culture.

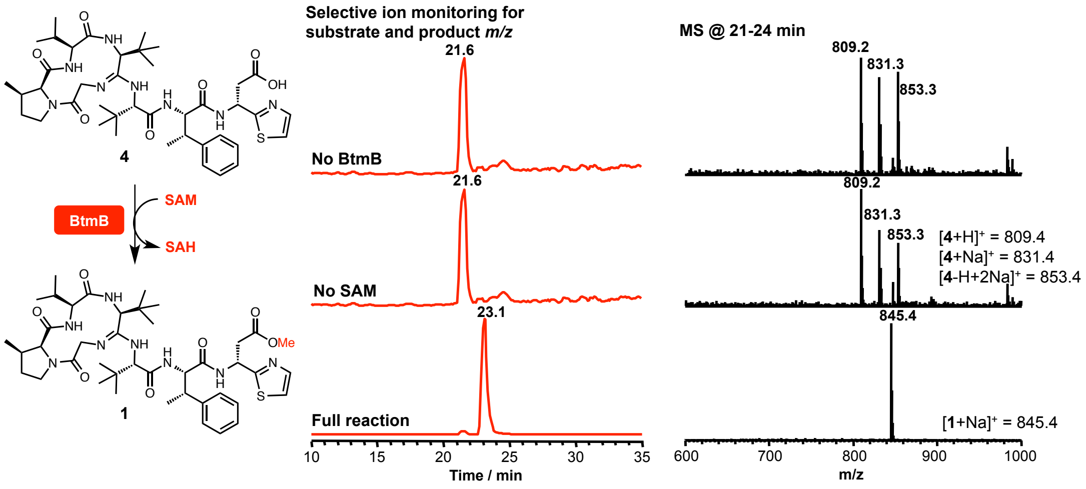

Figure S28 *In vitro* BtmB activity with **4** (*O*-desmethyl bottromycin A$_2$).

4. Supplementary References

[1] D. J. MacNeil, K. M. Gewain, C. L. Ruby, G. Dezeny, P. H. Gibbons, T. MacNeil, *Gene* **1992**, *111*, 61–68.

[2] W. Crone, F. J. Leeper, A. W. Truman, *Chem. Sci.* **2012**, *3*, 3516-3521.

[3] N. M. Vior, C. Olano, I. García, C. Méndez, J. A. Salas, *Microbiology* **2014**, *160*, 467–478.

[4] L. T. Fernández-Martínez, J. P. Gomez-Escribano, M. J. Bibb, *Mol. Microbiol.* **2015**, *97*, 502–514.

[5] Y. Kobayashi, M. Ichioka, T. Hirose, K. Nagai, A. Matsumoto, H. Matsui, H. Hanaki, R. Masuma, Y. Takahashi, S. Ōmura, et al., *Bioorg. Med. Chem. Lett.* **2010**, *20*, 6116–6120.

[6] M. S. Paget, L. Chamberlin, A. Atrih, S. J. Foster, M. J. Buttner, *J. Bacteriol.* **1999**, *181*, 204–211.
